# Alternative splicing of uromodulin enhances mitochondrial metabolism for adaptation to stress in kidney epithelial cells

**DOI:** 10.1172/JCI183343

**Published:** 2025-04-08

**Authors:** Azuma Nanamatsu, George J. Rhodes, Kaice A. LaFavers, Radmila Micanovic, Virginie Lazar, Shehnaz Khan, Daria Barwinska, Shinichi Makino, Amy Zollman, Ying-Hua Cheng, Emma H. Doud, Amber L. Mosley, Matthew J. Repass, Malgorzata M. Kamocka, Aravind Baride, Carrie L. Phillips, Katherine J. Kelly, Michael T. Eadon, Jonathan Himmelfarb, Matthias Kretzler, Robert L. Bacallao, Pierre C. Dagher, Takashi Hato, Tarek M. El-Achkar

**Affiliations:** 1 Department of Medicine, Division of Nephrology and Hypertension,; 2 Department of Anatomy, Cell Biology and Physiology, Medicine,; 3 Department of Biochemistry and Molecular Biology,; 4 Center for Proteome Analysis,; 5 Center for Computational Biology and Bioinformatics,; 6 Cellular Response Technologies Core, and; 7 Department of Pathology & Laboratory Medicine, Indiana University School of Medicine, Indianapolis, Indiana, USA.; 8 Indianapolis Roudebush VA Medical Center, Indianapolis, Indiana, USA.; 9 Department of Medicine, Mount Sinai School of Medicine, New York, New York, USA.; 10 Department of Medicine, University of Michigan, Ann Arbor, Michigan, USA.; 11 Department of Medical and Molecular Genetics, Indiana University School of Medicine, Indianapolis, Indiana, USA.

**Keywords:** Cell biology, Nephrology, Hypoxia, Mitochondria, Protein traffic

## Abstract

In the kidney, cells of thick ascending limb of the loop of Henle (TAL) are resistant to ischemic injury, despite high energy demands. This adaptive metabolic response is not fully understood even though the integrity of TAL cells is essential for recovery from acute kidney injury (AKI). TAL cells uniquely express uromodulin, the most abundant protein secreted in healthy urine. Here, we demonstrate that alternative splicing generates a conserved intracellular isoform of uromodulin, which contributes to metabolic adaptation of TAL cells. This splice variant was induced by oxidative stress and was upregulated by AKI that is associated with recovery, but not by severe AKI and chronic kidney disease (CKD). This intracellular variant was targeted to the mitochondria, increased NAD^+^ and ATP levels, and protected TAL cells from hypoxic injury. Augmentation of this variant using antisense oligonucleotides after severe AKI improved the course of injury. These findings underscore an important role of condition-specific alternative splicing in adaptive energy metabolism to hypoxic stress. Enhancing this protective splice variant in TAL cells could become a therapeutic intervention for AKI.

## Abstract 2

Kidney cells can protect themselves during injury by producing an isoform of the protein uromodulin that re-routes to the mitochondria to enhance energy production

## Graphical abstract

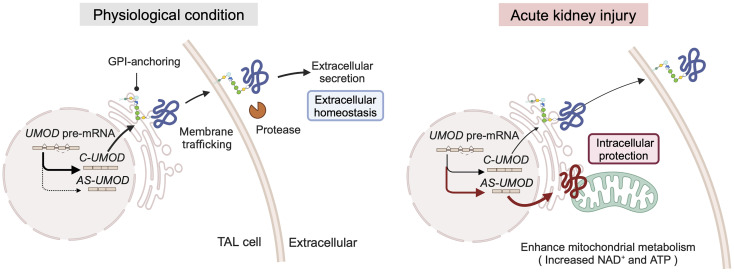

## Introduction

The kidney is one of the most energy-requiring organs (1, 2) with the second highest mitochondrial content and oxygen consumption following the heart (3, 4). This high energy consumption is needed for active transport mechanisms, contributing to urinary concentration and maintaining body hemostasis. Thick ascending limbs of the loop of Henle (TAL) cells, spanning from the medulla to cortex, are one of the most abundant epithelial cell types second to proximal tubule (PT) cells in the kidney. TAL cells have been reported to comprise approximately 20% of the cells in the kidney (5). TAL segments have high energy requirements for sodium reabsorption through the Na-K-Cl cotransporter (NKCC2), which is coupled to sodium-potassium ATPase. Despite these high energy demands, TAL cells remain histologically preserved (6) and retain higher levels of ATP (7) compared with PT cells in experimental models of hypoxic acute injury. How TAL cells preserve energy generation during injury is not fully understood, although several mechanisms have been proposed (7). In addition, the integrity of TAL cells is now recognized as a prognostic factor during kidney injury and repair by recent single-cell and spatial analysis (8, 9), clinical observations of TAL-specific molecules (10, 11) and an experimental model of TAL-specific injury (12). Therefore, protecting TAL cells from ischemic injury is important for improved recovery and outcomes.

Uromodulin (UMOD, also known as Tamm-Horsfall protein, gene name: *UMOD*) is a highly conserved and abundant secretory protein in the kidney and is mainly expressed in TAL cells (13, 14). The *UMOD* gene is composed of 11 exons, with exons 2 through 11 forming the protein-coding region. The exon 10 encoding region harbors a glycosylphosphatidylinositol-anchoring (GPI-anchoring) site. Since the GPI anchor is a major apical sorting signal (15), the UMOD protein is predominantly localized at the apical membrane. Membrane-bound UMOD positively regulates the activities of transporters, including NKCC2 (16–18). Membrane-bound UMOD is then cleaved by a protease hepsin (19) and released into the urine as the most abundant urinary protein in healthy individuals. Urinary UMOD maintains urinary tract health by preventing infections and kidney stone formation (13). The *UMOD* gene is strongly associated with kidney disease in genome-wide association studies (20), thereby suggesting a modulatory role in injury. Increasing clinical evidence supports a protective role of UMOD in AKI, where higher UMOD expression is linked to better renal outcomes (21–23). Consistent with these observations, we have shown in a knockout mouse model that UMOD deficiency aggravates AKI and impairs recovery (24–26). We have demonstrated that a fraction of UMOD is secreted into the interstitium and circulation, contributing to AKI recovery through its immunomodulatory effects (24, 26, 27). Therefore, enhancing our understanding of UMOD physiology will be beneficial for developing therapies that accelerate improved recovery from kidney injury.

Despite a large interest in the *UMOD* gene and the regulators of its expression, alternative splicing of *UMOD* and its relevance in disease are not well understood. Alternative splicing expands the diversity of proteins with multiple subcellular localizations and functions (28–30). Here, we identified a conserved alternatively spliced variant of *UMOD* that skips exon 10, thereby lacking the GPI-anchoring site. This isoform was induced by ischemic stress and is targeted intracellularly to the mitochondria, where it enhanced mitochondrial energy generation and preserved the integrity of TAL cells during injury. We also identified antisense oligonucleotides (ASOs) that enhance this variant and demonstrated their efficacy in AKI. These findings will expand our understanding of how epithelial cells adapt to stress through alternative splicing.

## Results

### Identification of alternatively spliced UMOD.

**Figure 1:**
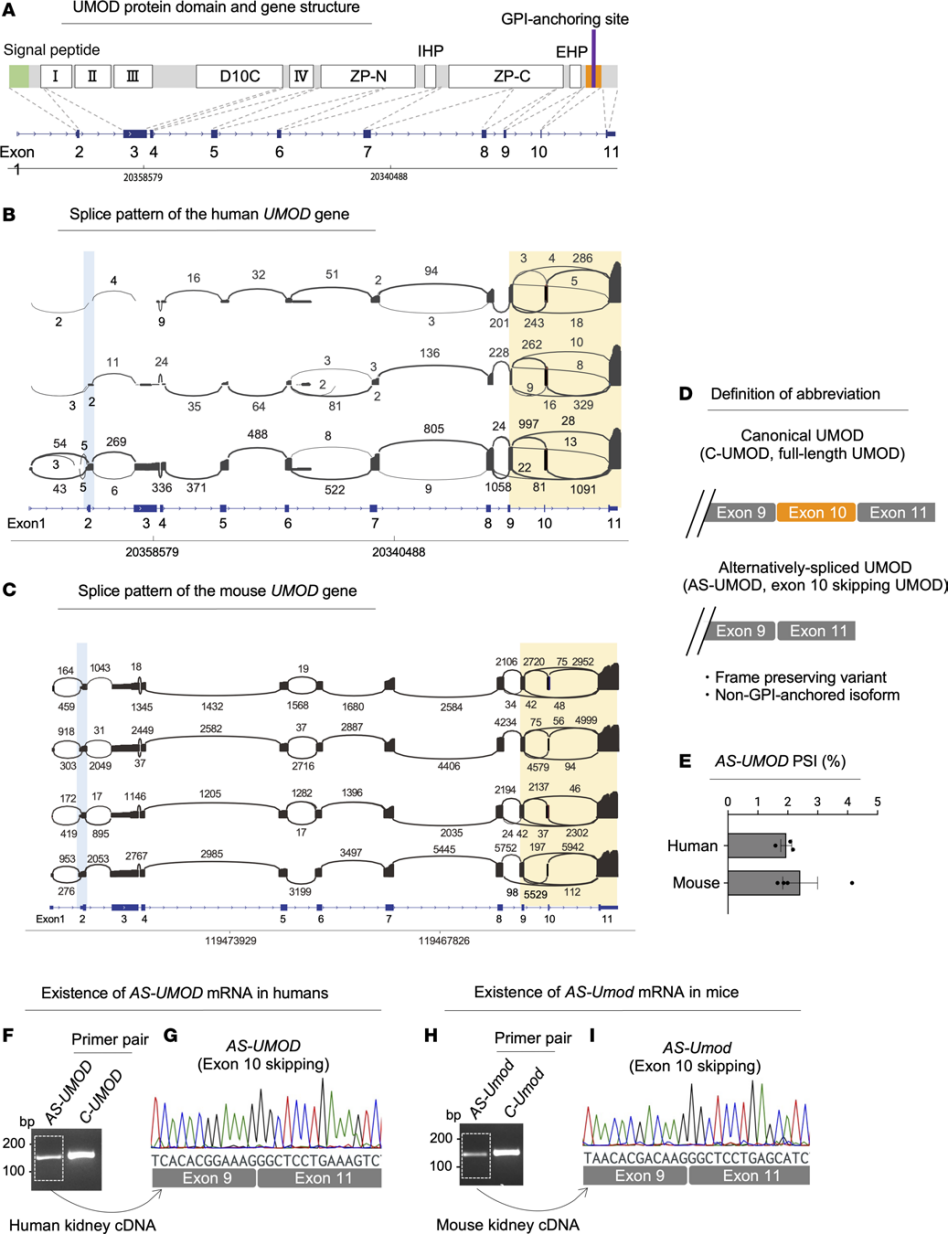
Identification of AS-UMOD. (**A**) The upper panel shows the primary structure and domains of UMOD. The 4 EGF-like domains are represented by the Roman numerals I through IV. D10C, domain with conserved 10 cysteines; ZP, zona pellucida; IHP, internal hydrophobic patch; EHP, external hydrophobic patch. The lower panel shows the exon/intron structure of the *UMOD* gene from Refseq (NCBI database). (**B** and **C**) Sashimi plot visualizes differentially spliced exons of the *UMOD* transcript isolated from (**B**) human and (**C**) mouse kidneys. Each numeral on the semicircle represents the number of RNA-Seq reads. Reads indicating alternative splicing sites of exon 2 and exon 10 skipping were highlighted in blue and yellow, respectively. *n* = 3 for human and *n* = 4 for mouse kidneys. (**D**) Definition of abbreviation. (**E**) Percent-splice-in (PSI) value of *AS-UMOD* calculated from Nanopore long-read RNA-Seq data (*n* = 3 for human and *n* = 4 for mouse kidneys). (**F**) RT-PCR for *AS-UMOD* and *C-UMOD* from human kidney cDNA. (**G**) RT-PCR product of **F** was purified and subsequent Sanger sequencing confirmed the existence of *AS-UMOD* (exon 10 skipping *UMOD*) in human kidneys. (**H**) RT-PCR for *AS-Umod* and *C-Umod* from mouse kidney cDNA. (**I**) RT-PCR product of **H** was purified and subsequent Sanger sequencing confirmed the existence of *AS-Umod* (exon 10 skipping *Umod*) in mouse kidneys. Data are represented as mean ± SEM.

Figure 1A shows the domains and gene structure of *UMOD*. The most common form of alternative splicing is cassette exons, where internal exons can either be included or excluded. To explore cassette exons in the *UMOD* gene, we screened differentially spliced exons in the *UMOD* gene using Nanopore long-read RNA-Seq of the human and mouse kidneys (Figure 1, B and C). We consistently found exon 10 skipping reads in both human and mouse kidneys. RNA-Seq data suggested multiple patterns of exon 10 skipping variants, including variants which additionally involve alternative 5′/3′ splicing sites or exon 9 skipping. We focused on the exon 10 skipping variant without any other alteration because this was (a) the only variant confirmed across all human (*n* = 3) and mouse (*n* = 4) samples and (b) a variant that restores the reading frame since exon 10 is composed of multiples of 3 nucleotides (39 nt in humans and 42 nt in mice) (Supplemental Figure 1A; supplemental material available online with this article; https://doi.org/10.1172/JCI183343DS1). The conservation of this splice variant in both humans and mice indicates its functional importance (31, 32). Moreover, frame preservation suggests that the transcript will likely be translated into a protein without degradation by nonsense-mediated mRNA decay (NMD) (33). Since exon 10 encodes the GPI-anchoring site (Figure 1A), its absence is likely to alter the subcellular localization of UMOD protein, leading to a distinct function. We defined exon 10–retaining UMOD (full-length UMOD) as canonical UMOD (C-UMOD) and exon 10–skipping UMOD as alternatively spliced UMOD (AS-UMOD) (Figure 1D). In our data, approximately 2%–3% of *UMOD* transcripts underwent exon 10 skipping (Figure 1E). Considering that *UMOD* is the most abundant transcript in the kidney (34), the relatively low percentage of *AS-UMOD* still constitutes a substantial abundance. Another remarkable and consistent splice variant both in humans and mice was the competing splice acceptor sites of exon 2 of the *UMOD* gene (Figure 1, B and C). This splicing event happens before the start of the coding sequence (CDS) and therefore will alter the 5′ UTR composition but not the CDS of the *UMOD* gene (Supplemental Figure 1B). The alteration of the 5′ UTR composition might affect the translation efficacy of the *UMOD* gene and therefore might have a biological relevance, although we did not conduct further analysis in this study.

To validate and quantitate *AS-UMOD* mRNA expression, we designed specific PCR primers for *C-UMOD* and *AS-UMOD* (Supplemental Figure 2A). Their specificity was validated by quantitative PCR (qPCR) using cDNA from cells which lack endogenous *UMOD* expression but overexpress one of the isoforms (Supplemental Figure 2, B and C). We then confirmed the presence of *AS-UMOD* mRNA in human and mouse kidneys by reverse transcription PCR (RT-PCR) and subsequent Sanger sequencing (Figure 1, F–I).

### Acute kidney injury induces AS-UMOD expression.

Given the association between UMOD and acute kidney injury (AKI), we determined whether AS-UMOD expression is altered in AKI. We utilized a murine renal ischemia-reperfusion injury (IRI) model, a well-established model of AKI (35). In the renal IRI model, clamp time of the renal vascular pedicles is the major determinant of AKI severity (35) and allows the establishment of 2 different models (25, 36): mild IRI (22-minute clamp time) with rapid recovery likely due to adaptive repair, and severe IRI (30-minute clamp time) with delayed recovery likely due to maladaptive repair.

**Figure 2:**
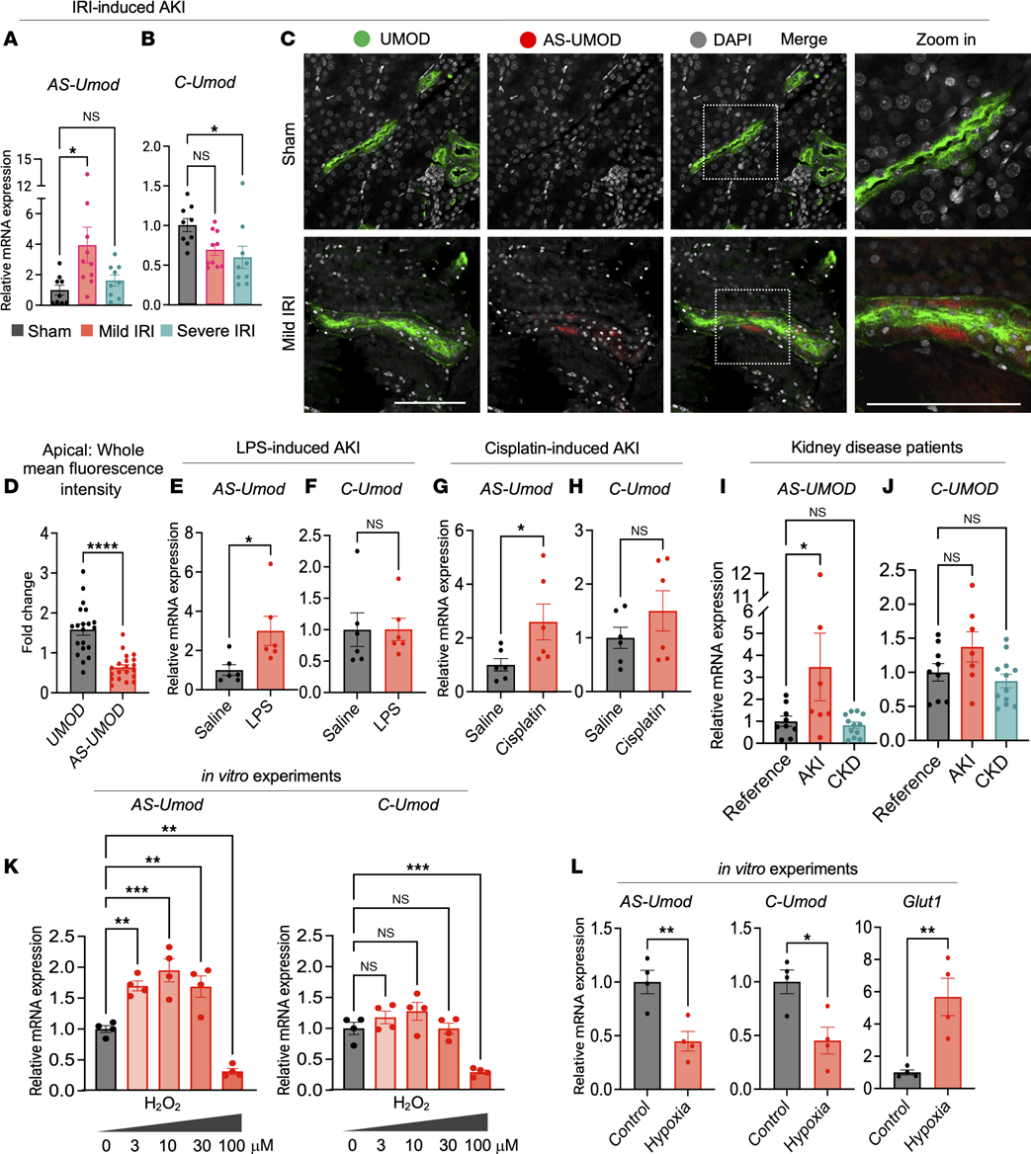
AKI induces AS-UMOD expression. (**A** and **B**) Relative mRNA expression of *AS-Umod* and *C-Umod* normalized to *Gapdh* in IRI mice. WT mice underwent sham, mild IRI, or severe IRI surgery and were harvested 24 hours after the surgery. *n* = 9–10 per group. (**C**) Immunofluorescence of subcortical region of murine kidneys 24 hours after the surgery. *n* = 5 mice per group. Scale bars: 100 μm. (**D**) Apical membrane localization of UMOD and AS-UMOD, determined by the ratio of apical membrane: whole tubules mean signal intensity was quantified using ImageJ (NIH). *n* = 20 tubules from 5 mild IRI kidneys for each group. (**E** and **F**) Relative mRNA expression of *AS-Umod* (**E**) and *C-Umod* (**F**) normalized to *Gapdh* in LPS-induced AKI mice. 5 mg/kg LPS was injected via intraperitoneal injection and mice were harvested 24 hours after injection. *n* = 6 per group. (**G** and **H**) Relative mRNA expression of *AS-Umod* (**G**) and *C-Umod* (**H**) normalized to *Gapdh* in cisplatin-induced AKI mice. 20 mg/kg cisplatin was injected via intraperitoneal injection and mice were harvested 72 hours after injection. *n* = 6 per group. (**I** and **J**) Relative mRNA expression of *AS-UMOD* (**I**) and *C-UMOD* (**J**) normalized to *NKCC2* in human kidney samples from the KPMP. *n* = 7–12 per group. (**K**) Relative mRNA expression of *AS-Umod* and *C-Umod* normalized to *Gapdh* in MKTAL cells treated with various concentrations of hydrogen peroxide (H_2_O_2_) for 6 hours. *n* = 4 per group. (**L**) Relative mRNA expression of *AS-Umod*, *C-Umod*, and *Glut1* normalized to *Hprt* in hypoxia conditions. MKTAL cells were cultured in control (normoxia) or hypoxia conditions for 6 hours. *n* = 4 per group. Data were analyzed by unpaired *t* test (between 2 conditions, **D**–**H**, and **L**) or 1-way ANOVA with embedded comparisons between 2 individual groups (among multiple conditions, **A**, **B**, and **I**–**K**) and are represented as mean ± SEM. *P < 0.05; **P < 0.01; ***P < 0.001; ****P < 0.0001.

qPCR analysis showed that mild IRI upregulates *AS-Umod* mRNA expression (Figure 2A), but not *C-Umod* mRNA (Figure 2B), indicating a unique injury-related induction of *AS-Umod*. Interestingly, severe IRI failed to upregulate *AS-Umod* (Figure 2A). To evaluate AS-UMOD expression at the protein levels, we raised AS-UMOD–specific antibodies, in which the epitope is located on the boundary of UMOD exons 9 and 11 and does not react with the exon 10–containing peptide (Supplemental Figure 3, A and B). We also confirmed that commercial UMOD antibodies used in this study recognize both C-UMOD and AS-UMOD by characterizing their epitopes based on the manufacturer’s information and epitope mapping (Supplemental Figure 3, C and D). Immunofluorescence analysis using these antibodies demonstrated that AS-UMOD is detectable at the protein levels in TAL cells after mild IRI (Figure 2C). *Umod^–/–^* mice did not show detectable signals even after mild IRI, indicating the specificity of UMOD and AS-UMOD antibodies (Supplemental Figure 3E). AS-UMOD induction was observed more frequently in cortical and subcortical TAL cells than medullary TAL cells (Supplemental Figure 4, A and B). Notably, AS-UMOD was localized in the cytoplasm of TAL cells, while (total) UMOD, predominantly composed of C-UMOD, was localized mainly at the apical membrane (Figure 2, C and D).

To assess whether AS-UMOD induction is a common observation in AKI, we developed LPS- and cisplatin-induced AKI models (Supplemental Figure 5, A–D). LPS and cisplatin (single dose administration) cause remarkable proximal tubular injury (35, 37) and can induce stress to TAL cells (38, 39). In both these models, recovery typically occurs after AKI (40, 41). We found that *AS-Umod* but not *C-Umod* mRNA expression was upregulated both in LPS and cisplatin models (Figure 2, E–H), which is similar to mild IRI. These results indicate that AS-UMOD induction is not specific to ischemia-reperfusion but common to AKI that is associated with recovery.

We next evaluated whether AS-UMOD induction is observed in chronic kidney disease (CKD) models. *AS-Umod* mRNA expression was not altered in 8 weeks after severe IRI, which showed elevated urea levels and *Col1a1* and *Fibronectin1* mRNA expression when using 129/SvEv background mice (Supplemental Figure 5, E and F). Similarly, the aristolochic acid nephropathy model, which caused elevated urea levels and *Col1a1* and *Fibronectin1* mRNA expression at 4 weeks after injection, did not induce *AS-Umod* mRNA expression (Supplemental Figure 5, G and H).

Furthermore, we assessed the dynamics of AS-UMOD in human kidney disease using specimens from the Kidney Precision Medicine Project (KPMP). Summary demographics of reference and disease kidney tissue specimens are presented in Supplemental Table 1. In line with the findings from mouse models, *AS-UMOD* mRNA expression was higher in AKI but not in CKD patients compared with healthy reference (Figure 2, I and J).

In summary, AS-UMOD induction commonly occurs in various forms of AKI associated with recovery. This isoform is not induced in severe IRI, which can be associated with maladaptive repair, or in CKD. AS-UMOD was an intracellular isoform of UMOD and was mainly induced in the cortical region.

### Oxidative stress induces AS-UMOD expression.

To understand the molecular mechanism governing the splicing of the *UMOD* gene, we utilized MKTAL cells, an immortalized mouse-derived TAL cell line which expresses endogenous UMOD (42, 43). We hypothesized that AS-UMOD expression is regulated by oxidative stress, since it is one of the common pathogenesis of IRI-, LPS-, and cisplatin-induced AKI (35) and is linked to alternative splicing (44). We found that 3–30 μM hydrogen peroxide induced *AS-Umod* mRNA but not *C-Umod* mRNA expression while higher concentrations of hydrogen peroxide decreased *AS-Umod* mRNA expression (Figure 2K). These findings suggest that mild but not severe oxidative stress drives alternative splicing of UMOD, consistent with in vivo observation where mild but not severe IRI induced AS-UMOD expression (Figure 2A).

We also tested the effect of hypoxia by itself without reoxygenation on AS-UMOD induction. Hypoxia did not induce *AS-Umod* mRNA expression, but rather inhibited it (Figure 2L). We verified successful induction of hypoxia by increased *Glut1* mRNA expression (45) and we used *Hprt* as a housekeeping gene for the hypoxia experiment since hypoxia elevates *Gapdh* mRNA expression (46).

### AS-UMOD is a cytoprotective intracellular isoform of UMOD.

To investigate the intracellular relevance of AS-UMOD, we utilized Madin-Darby canine kidney (MDCK) renal epithelial cells overexpressing UMOD, a well-established model for studying UMOD biology, including intracellular trafficking (47, 48), proteolytic cleavage (19), and extracellular secretion (47, 49). Another advantage of MDCK cells is that they do not express endogenous UMOD, eliminating any concerns of interference from the latter.

**Figure 3:**
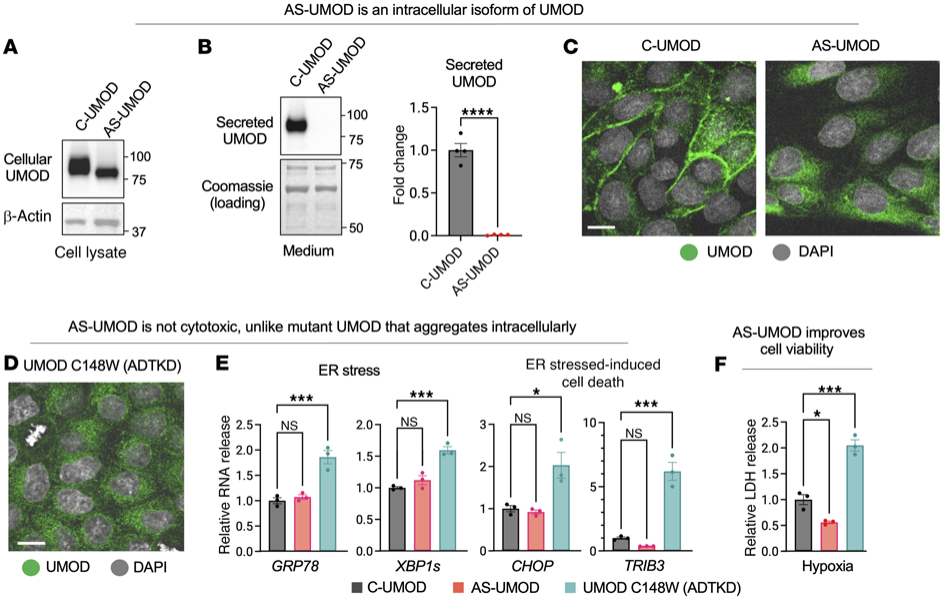
AS-UMOD is a cytoprotective intracellular isoform of UMOD. MDCK cells stably expressing C-UMOD or AS-UMOD were established by lentiviral transduction. (**A** and **B**) Immunoblotting of UMOD in MDCK cell lysate and medium, respectively. Coomassie staining was used as a loading control for medium. *n* = 4. (**C**) Immunofluorescence of C-UMOD and AS-UMOD in MDCK cells. *n* = 3. Scale bar: 10 μm. (**D**) Immunofluorescence of UMOD C148W, an ADTKD-causing mutant in MDCK cells. *n* = 3. Scale bar: 10 μm. (**E**) Relative mRNA expression of ER stress–related genes normalized to *GAPDH* expression. *n* = 3. (**F**) LDH assay in MDCK cells. MDCK cells were cultured in normoxia or hypoxia conditions for 6 hours. LDH concentration in the media was measured and normalized to total cell number. *n* = 3. Data were analyzed by unpaired *t* test (between 2 conditions, **B**) or 1-way ANOVA with embedded comparisons between 2 individual groups (among multiple conditions, **E** and **F**) and are represented as mean ± SEM. *P < 0.05; ***P < 0.001; ****P < 0.0001.

MDCK cells stably expressing human C-UMOD or AS-UMOD were established by lentiviral transduction. We first confirmed that *UMOD* mRNA expression was comparable in both cell lines by using qPCR primers targeted *UMOD* exon 6 to detect both isoforms (Supplemental Figure 6A). Immunoblotting indicated that AS-UMOD had a lower molecular weight and was not secreted extracellularly, whereas C-UMOD was released into the medium (Figure 3, A and B). Consistent with these findings, immunofluorescence analysis showed that AS-UMOD is localized intracellularly, while C-UMOD is predominantly localized at the plasma membrane (Figure 3C). These results are in line with the intracellular localization of AS-UMOD in mouse kidneys (Figure 2, C and D) and further supports that AS-UMOD is an intracellular isoform UMOD.

We next investigated whether this variant could be cytoprotective or cytotoxic. Mutations in the *UMOD* gene cause intracellular localization of UMOD protein and drive one of the most common familial forms of kidney failures, autosomal dominant tubulointerstitial kidney disease (ADTKD) (50). Mutant UMOD misfolds, accumulates intracellularly, induces endoplasmic reticulum (ER) stress, and finally leads to interstitial fibrosis (50). We hypothesized that AS-UMOD behaves differently from ADTKD-causing mutant UMOD, because mutations mostly occur in the N-terminal half of UMOD (13), relatively far from exon 10 (Figure 1A). To test this, we studied MDCK cells stably expressing UMOD C148W, a representative ADTKD-causing mutation. In line with previous studies (48, 51–53), the ADTKD-causing mutant was localized intracellularly (Figure 3D) and triggered ER stress (*GRP78* and *XBP1s*) and ER stress–induced cell death (*CHOP* and *TRIB3*) gene expression (Figure 3E). Unlike UMOD C148W, AS-UMOD did not induce this stress response (Figure 3E). Moreover, in contrast to UMOD C148W mutant, AS-UMOD improved cell viability in hypoxia conditions compared with C-UMOD (Figure 3F). These results suggest that AS-UMOD is a cytoprotective intracellular isoform of UMOD, unlike the disease-causing mutant UMOD.

### AS-UMOD partially localizes to the mitochondria and enhances mitochondrial energy generation.

**Figure 4:**
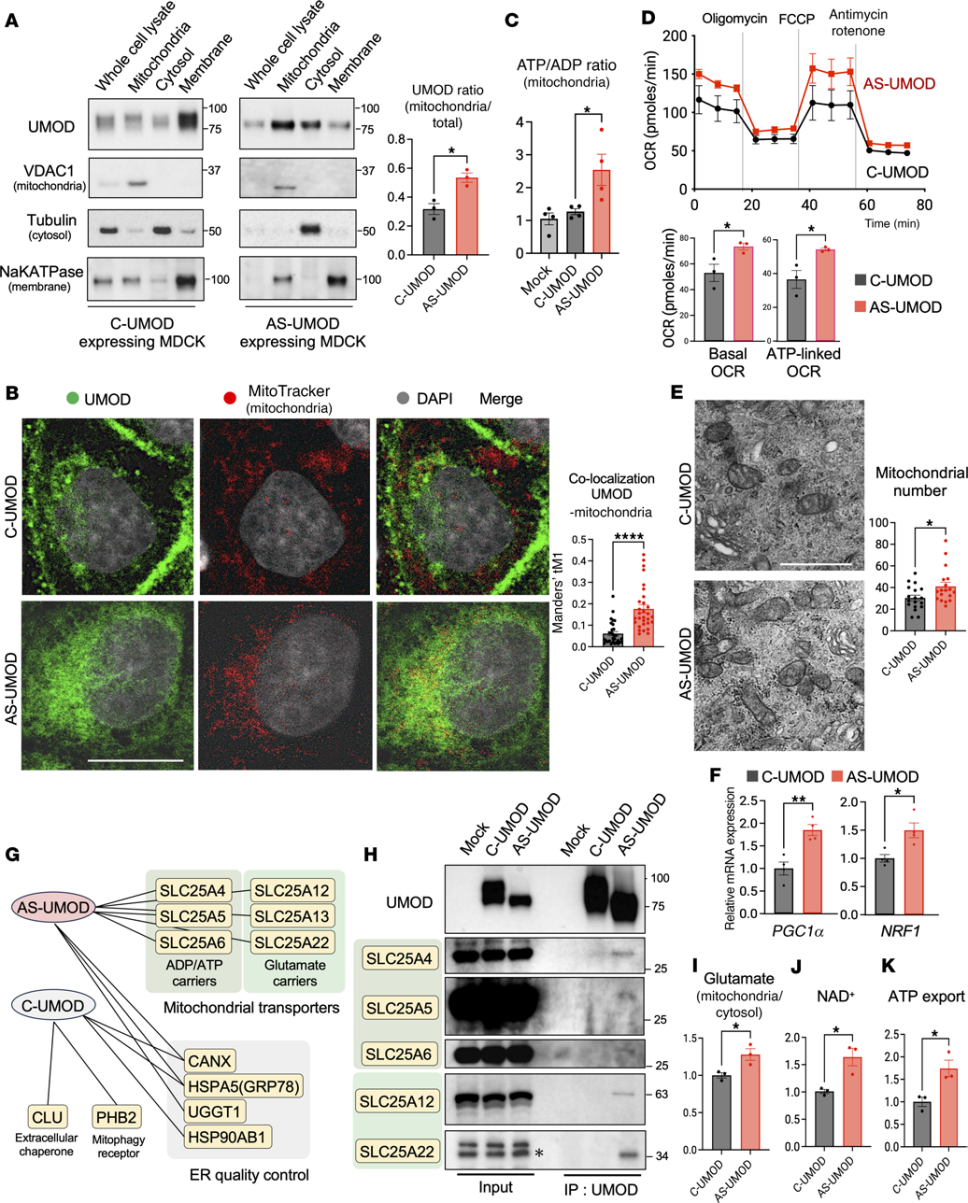
AS-UMOD enhances mitochondrial energy generation. (**A**) Immunoblotting of MDCK cells expressing C-UMOD or AS-UMOD after subcellular fractionation. The same amount of protein was applied for each fraction. The ratio of mitochondrial/total (mitochondrial, cytosolic, and membrane) UMOD expression was quantified by densitometry analysis. *n* = 3. (**B**) Immunofluorescence of MDCK cells expressing C-UMOD or AS-UMOD. Colocalization analysis between UMOD and mitochondria (Mitotracker) in MDCK cells. Manders’ tM1 represents a fraction of UMOD overlapping with mitochondria. *n* = 30 cells per group from 3 independent experiments. Scale bar: 10 μm. (**C**) ATP/ADP ratio of mitochondria isolated from MDCK cells. *n* = 4. (**D**) Mitochondrial respiration measurement in MDCK cells expressing C-UMOD or AS-UMOD using Seahorse. OCR, oxygen consumption rate; FCCP, carbonyl cyanide *p*-trifluoromethoxyphenylhydrazone. *n* = 3. (**E**) Transmission electron microscopy in MDCK cells expressing C-UMOD or AS-UMOD. Scale bar: 1 μm. Mitochondrial number per 100 μm^2^ cell area (excluding nucleus) was quantitated. *n* = 18 cells for each group from 2 independent experiments. (**F**) Relative mRNA expression of *PGC1α* and *NRF1* normalized to *GAPDH*. *n* = 4. (**G**) Interactome map of C-UMOD and AS-UMOD in MDCK cells obtained from AP-MS analysis. (**H**) Coimmunoprecipitation with anti-UMOD antibody to validate the AP-MS analysis. Asterisk indicates lower band is the target band for SLC25A22. *n* = 2. (**I**) The ratio of mitochondrial/cytosolic glutamate levels in MDCK cells. *n* = 3. (**J**) NAD^+^ levels normalized to protein concentration. *n* = 3. (**K**) ADP/ATP carrier-mediated ATP export after ADP addition to the isolated mitochondria from MDCK cells. *n* = 3. Data were analyzed by unpaired *t* test (between 2 conditions, **A**, **B**, **D**–**F**, and **I**–**K**) or 1-way ANOVA with embedded comparisons between 2 individual groups (among multiple conditions, **C**) and are represented as mean ± SEM. *P < 0.05; **P < 0.01; ****P < 0.0001.

To explore the cytoprotective mechanism of this intracellular isoform, AS-UMOD, we investigated its intracellular localization. Immunoblotting of subcellular fractions isolated by differential centrifugation (54, 55) (Figure 4A) and immunofluorescence analysis (Figure 4B) suggested that AS-UMOD is, at least partially, localized to the mitochondria, and this is a distinct localization from C-UMOD. We next examined the effect of AS-UMOD on mitochondrial function. AS-UMOD increased both the ATP/ADP ratio in mitochondria (Figure 4C) and the oxygen consumption rate (Figure 4D), indicating that AS-UMOD enhances mitochondrial ATP generation.

To assess how AS-UMOD upregulates mitochondrial function, we studied its effect on mitochondrial biogenesis. AS-UMOD slightly but significantly increased mitochondrial number (Figure 4E), *PGC1*α (*PPARGC1A*), and *NRF1* mRNA expression (Figure 4F), and mitochondrial protein (TOM20 and TIM23) expression (Supplemental Figure 6B). These results suggest that AS-UMOD could enhance mitochondrial biogenesis, thereby contributing, at least partially, to the increased ATP production.

### AS-UMOD is associated with SLC25 carriers.

To further understand how AS-UMOD enhances mitochondrial biogenesis and ATP generation, we performed affinity purification coupled to mass spectrometry (AP-MS) (Supplemental Figure 6C), a potent approach to determine the function of alternatively spliced variants (28). In line with its mitochondrial localization, AS-UMOD interactome showed enrichment of mitochondrial proteins compared with C-UMOD. Strikingly, all the unique mitochondrial interactors of AS-UMOD were members of the mitochondrial solute carrier family 25 (SLC25), aspartate glutamate carrier (SLC25A12, SLC25A13), glutamate carrier (SLC25A22), and ADP/ATP carrier (SLC25A4, SLC25A5, SLC25A6) (Figure 4G). We confirmed the interaction between AS-UMOD and SLC25 carriers by coimmunoprecipitation (Figure 4H). AS-UMOD also interacted with ER-resident proteins, which are responsible for ER quality control. Among these, calnexin (CANX) and GRP78 (HSPA5) were common interactors for AS-UMOD and C-UMOD. Note that CANX and GRP78 have previously been identified as interactors of C-UMOD (WT UMOD) by other groups (51, 52), supporting the fidelity of our AP-MS analysis.

SLC25 carriers are crucial for mitochondrial energy generation, acting by transporting metabolites across the impermeable inner membrane (Supplemental Figure 6D) (56). Glutamate carrier imports glutamate into the mitochondria. Aspartate glutamate carrier is also responsible for the malate-aspartate shuttle (MAS), (57) which provides mitochondrial NADH, facilitating the electron transport chain (58) and subsequent generation of NAD^+^ (59). NAD^+^ is a rate-limiting coenzyme for mitochondrial function (60, 61) and considered to mutually activate with PGC1α (62). ADP/ATP carriers exchange cytosolic ADP and synthesized ATP (63). These carriers are abundantly expressed in mouse TAL cells based on previous RNA-Seq analysis of microdissected mouse tubular segments (Supplemental Figure 6E) (64) and single-cell RNA-Seq analysis of mouse kidneys (65, 66), except for SLC25A6.

### AS-UMOD and activation of SLC25 carriers.

We then evaluated the impact of AS-UMOD on SLC25 carrier activities. The ratio of mitochondrial/cytosolic glutamate was higher in AS-UMOD–expressing cells, which could support enhanced entry of glutamate into the mitochondria (Figure 4I). In addition, intracellular NAD^+^ levels were also higher in AS-UMOD–expressing cells (Figure 4J). ADP/ATP carrier activity, assessed by ATP export after external ADP addition to the mitochondria, was increased in AS-UMOD expression cells (Figure 4K).

Altogether, AS-UMOD, localized at the mitochondria, increases ATP production. The association between AS-UMOD and SLC25 carriers might contribute to increased glutamate utilization and enhanced MAS activity, NAD^+^ generation, mitochondrial biogenesis, and ATP export, which could lead to increased ATP generation.

### Generation of Umod exon 10 heterozygous knockout (Exon10^+/–^) MKTAL cell line.

**Figure 5:**
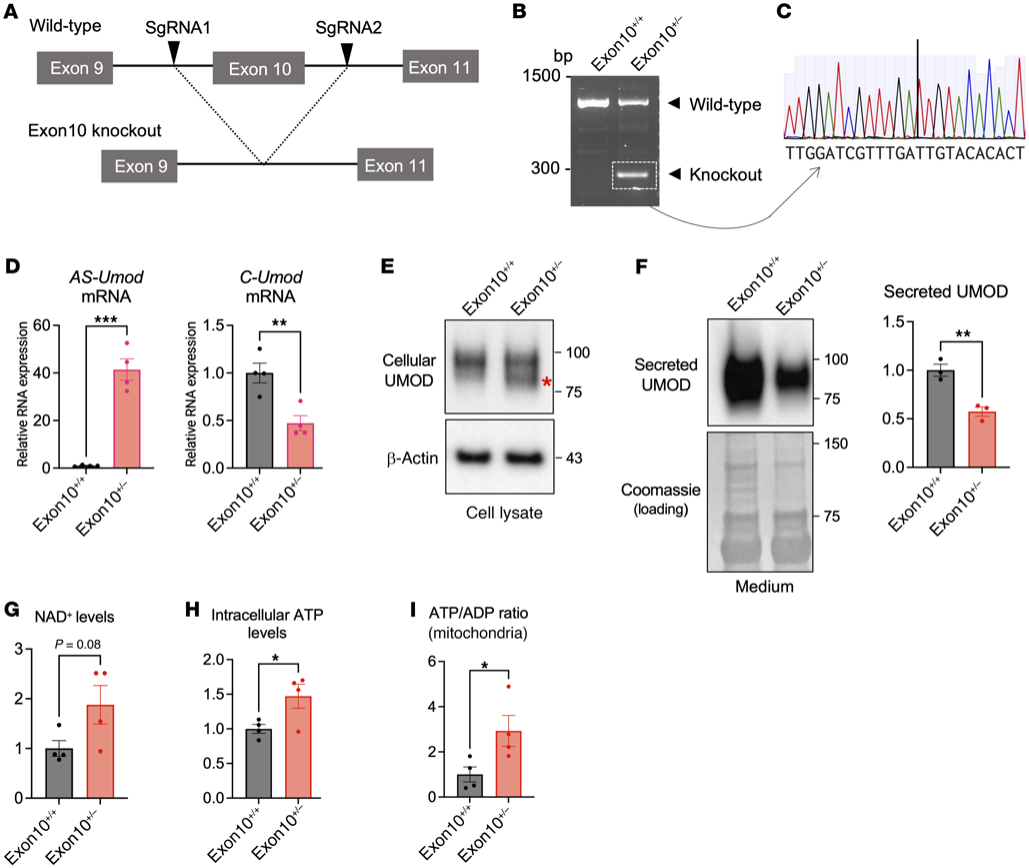
Generation of *Umod* exon10 heterozygous knockout (Exon10^+/–^) MKTAL cell line. (**A**) Two sgRNAs were designed to cut the intronic region around exon 10. (**B** and **C**) Genotyping PCR (**B**) and subsequent Sanger sequencing of the PCR product (**C**) confirmed successful heterozygous knockout of *Umod* exon 10. (**D**) Relative mRNA expression of *AS-Umod* and *C-Umod* normalized to *Gapdh* in WT (Exon10^+/+^) and *Umod* exon10 heterozygous knockout (Exon10^+/–^) MKTAL cells. *n* = 4. (**E** and **F**) Immunoblotting of UMOD in cell lysate (**E**) and medium (**F**). Red asterisk corresponds to AS-UMOD. Coomassie staining was used as a loading control for medium. *n* = 3. (**G**) NAD^+^ levels normalized to protein concentration. *n* = 4. (**H**) ATP levels normalized to protein concentration. *n* = 4. (**I**) ATP/ADP ratio of mitochondria isolated from Exon 10^+/+^ and Exon 10^+/–^ MKTAL cells. *n* = 4. Data were analyzed by unpaired *t* test and are represented as mean ± SEM. *P < 0.05; **P < 0.01; ***P < 0.001.

To further confirm the favorable metabolic role of AS-UMOD in TAL cells which express endogenous UMOD, we generated CRISPR/Cas9-mediated *Umod* exon 10 knockout MKTAL cells (Figure 5). We utilized heterozygous exon 10 knockout MKTAL cells in order to analyze the role of AS-UMOD in the existence of C-UMOD in TAL cells. We designed 2 sgRNA to cut the intronic region around exon 10 (Figure 5A). Successful heterozygous knockout of *Umod* exon 10 was confirmed by genotyping PCR (Figure 5B) and subsequent Sanger sequencing (Figure 5C). *AS-Umod* mRNA expression was approximately 40-fold higher in *Umod* exon 10 heterozygous knockout (exon10^+/–^) cells (Figure 5D). Immunoblotting of exon10^+/–^ cell lysate showed a lower molecular weight band, which corresponds to AS-UMOD (Figure 5E). Extracellular secretion of UMOD was decreased in exon10^+/–^ cells, consistent with the increased intracellular AS-UMOD and decreased secretory C-UMOD (Figure 5F). Intracellular NAD^+^ level was trending higher in exon10^+/–^ cells (Figure 5G). Intracellular ATP levels (Figure 5H) and the mitochondrial ATP/ADP ratio were increased in exon10^+/–^ cells (Figure 5I). These results are in line with the localization and the role of AS-UMOD, which have been shown by MDCK cells overexpressing AS-UMOD (Figures 3 and 4).

### AS-UMOD does not interact with C-UMOD in MDCK cells.

**Figure 6:**
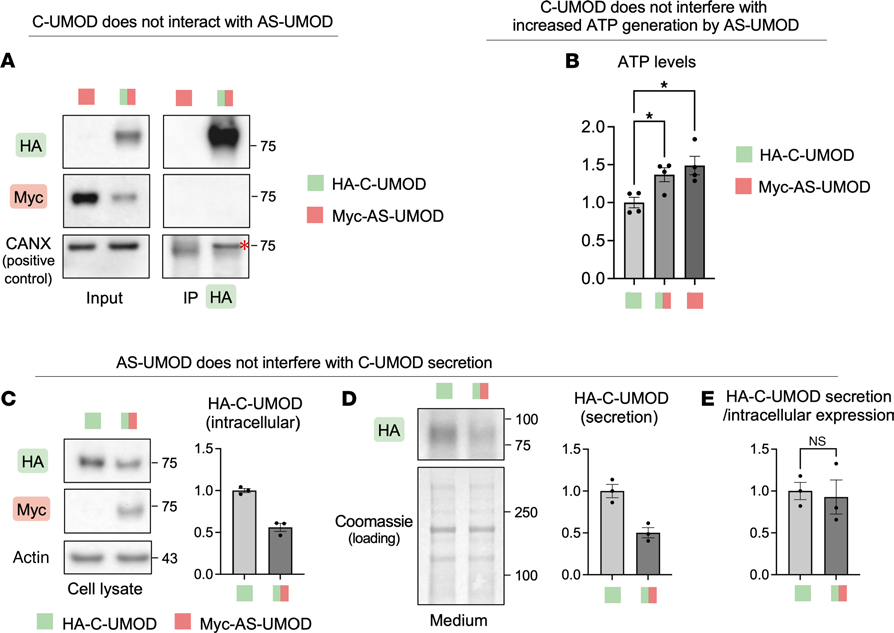
Cotransduction of C-UMOD and AS-UMOD. HA-C-UMOD and Myc-AS-UMOD were cotransduced to MDCK cells and their interaction was evaluated. (**A**) Coimmunoprecipitation with anti-HA antibody in MDCK cells expressing MYC-AS-UMOD only (lane 1) or an equal amount of HA-C-UMOD and Myc-AS-UMOD (lane 2). CANX was used as a positive control of coimmunoprecipitation. Red asterisk corresponds to CANX. *n* = 2. (**B**) Intracellular ATP levels normalized to protein concentration. *n* = 4. (**C** and **D**) Immunoblotting analysis of MDCK cells expressing HA-C-UMOD only (lane 1) or equal amount of HA-C-UMOD and Myc-AS-UMOD (lane 2). Densitometric analysis of HA-C-UMOD is presented. *n* = 3. (**E**) Secreted HA-C-UMOD normalized by intracellular HA-C-UMOD expression. *n* = 3. Data were analyzed by unpaired *t* test (between 2 conditions, **C**–**E**) or 1-way ANOVA with embedded comparisons between 2 individual groups (among multiple conditions, **B**) and are represented as mean ± SEM. *P < 0.05.

We next conducted cotransduction of Myc-tagged AS-UMOD and HA-tagged C-UMOD in MDCK cells to assess whether these 2 isoforms interact. Coimmunoprecipitation with anti-HA antibody using MDCK cells expressing only Myc-tagged human AS-UMOD (Myc-AS-UMOD) (100%) (negative control) or an equal amount of hemagglutinin (HA)-tagged human C-UMOD (HA-C-UMOD) (50%) and Myc-AS-UMOD (50%) did not show any interaction between the 2 isoforms (Figure 6A). CANX was used as a positive control for coimmunoprecipitation since it interacts with UMOD (Figure 4G) (51). In line with the lack of interaction between 2 isoforms, MDCK cells expressing only Myc-AS-UMOD (100%) as well as MDCK cells expressing both Myc-AS-UMOD (50%) and HA-C-UMOD (50%), showed higher intracellular ATP levels compared with MDCK cells expressing only HA-C-UMOD (100%) (Figure 6B). This suggests that increased ATP generation by AS-UMOD is not affected by C-UMOD. We next tested to determine whether AS-UMOD interferes with the extracellular secretion of C-UMOD using cells expressing only HA-C-UMOD (100%) or an equal amount of HA-C-UMOD (50%) and Myc-AS-UMOD (50%). As expected, intracellular HA-C-UMOD expression was approximately half in coexpression cells (Figure 6C). Secreted HA-C-UMOD in coexpression cells was also approximately half in coexpression cells (Figure 6D) and was equivalent to cells solely expressing HA-C-UMOD when normalized by lysate HA-C-UMOD abundance (Figure 6E). This suggests that AS-UMOD does not entrap the extracellular secretion of C-UMOD. This is in line with the data from MKTAL cells where extracellular secretion of UMOD was reduced to approximately 50% in heterozygous *Umod* exon 10 knockout cells (Figure 5F).

### Identification of splice-switching ASOs that induce AS-UMOD in MKTAL cells.

**Figure 7:**
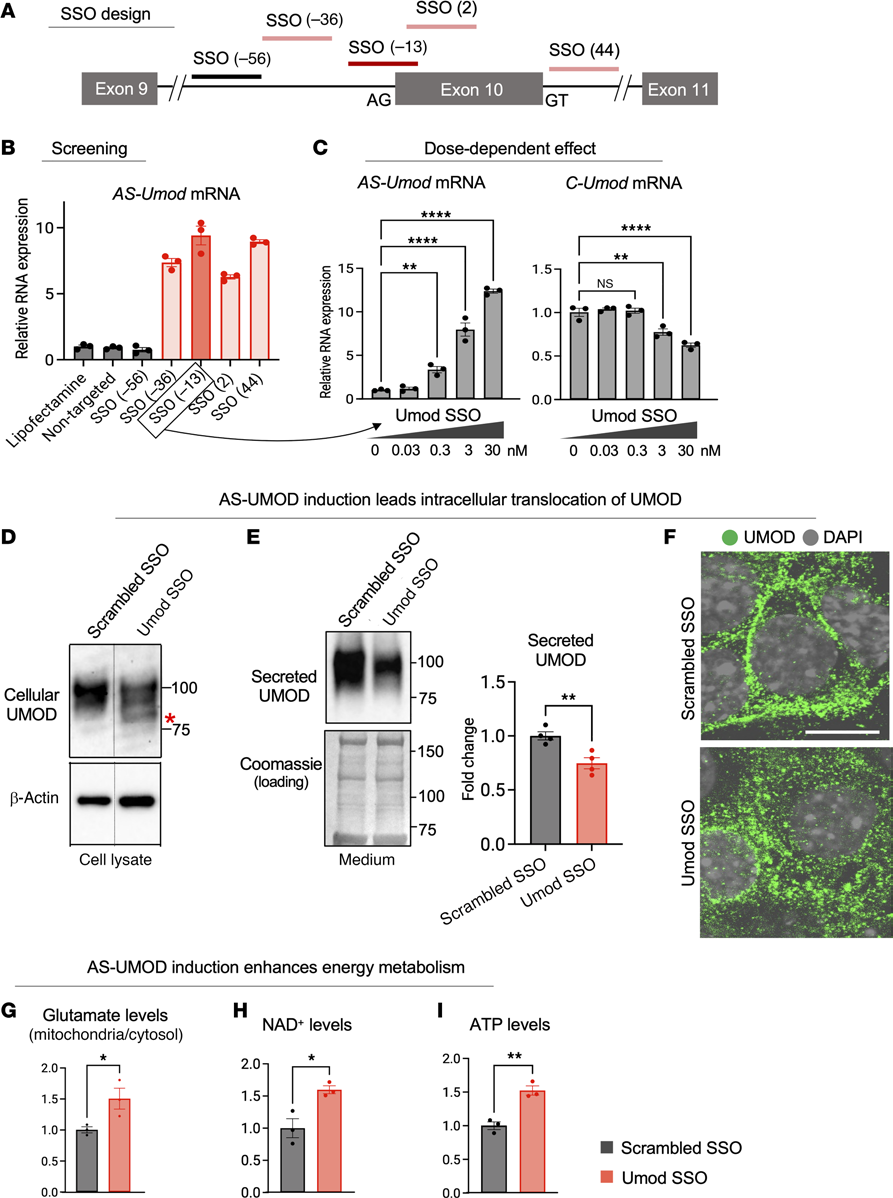
Identification of SSOs that induce AS-UMOD in MKTAL cells. (**A**) Design of SSOs to induce *AS-Umod* expression. Numbers in parentheses indicate position from the first base of exon 10. (**B**) Relative mRNA expression of *AS-Umod* normalized to *Gapdh* in MKTAL cells transfected with 30nM SSOs for 24 hours. Lipofectamine alone and nontargeted SSO were used as negative controls. *n* = 3. (**C**) Relative mRNA expression of *AS-Umod* and *C-Umod* normalized to *Gapdh* in MKTAL cells transfected with various concentrations of Umod SSO for 48 hours. Umod SSO corresponds to SSO (–13). *n* = 3. (**D**–**I**) MKTAL cells were treated with 30 nM scrambled SSO or Umod SSO for 48 hours. (**D** and **E**) Immunoblotting of UMOD in cell lysate and medium, respectively. Red asterisk corresponds to AS-UMOD. Coomassie staining was used as a loading control for medium. *n* = 4. (**F**) Immunofluorescence of UMOD. *n* = 2. Scale bar: 10 μm. (**G**) The ratio of mitochondrial/cytosolic glutamate levels. *n* = 3. (**H**) NAD^+^ levels normalized to protein concentration. *n* = 3. (**I**) ATP levels normalized to protein concentration. *n* = 3. Data were analyzed by unpaired *t* test (between 2 conditions, **E** and **G**–**I**) or 1-way ANOVA with embedded comparisons between 2 individual groups (among multiple conditions, **C**) and are represented as mean ± SEM. *P < 0.05; **P < 0.01; ****P < 0.0001.

Given the favored metabolic roles of AS-UMOD, we hypothesized that its induction during injury is beneficial. To this end, we leveraged splice-switching antisense oligonucleotides (SSOs) that induce AS-UMOD. SSOs are a type of ASO, which are defined as small, synthetic, single-stranded nucleic acid polymers that bind to a complementary sequence in the transcript and modulate gene expression. SSOs mask the splice sites and regulatory sequence, leading to exon skipping or inclusion (67, 68). We designed 5 SSOs that target exon 10 and surrounding introns including 3′ and 5′ splice sites (Figure 7A). We identified 4 SSOs that markedly induced endogenous *AS-Umod* mRNA expression in MKTAL cells (Figure 7B). We chose the most potent SSO (–13), which targets the 5′ splicing site (splice acceptor site) of exon 10, for subsequent analysis and named it Umod SSO. Umod SSO upregulated *AS-Umod* mRNA and downregulated *C-Umod* in a dose-dependent manner (Figure 7C). We also designed a scrambled SSO as a negative control for Umod SSO.

Immunoblotting of MKTAL cell lysate showed that AS-UMOD induction using Umod SSO generated a lower molecular weight band, which corresponds to AS-UMOD (Figure 7D), suggesting successful AS-UMOD protein expression, similar to the heterozygous exon 10 knockout MKTAL cells (Figure 5E). Immunoblotting of medium and immunofluorescence confirmed that Umod SSO leads to intracellular localization of UMOD (Figure 7, E and F), in line with the increased intracellular AS-UMOD and decreased membranous/secreted C-UMOD. Treatment of MKTAL cells with Umod SSO also increased mitochondrial glutamate (Figure 7G), intracellular NAD^+^ (Figure 7H), and ATP levels (Figure 7I), which are consistent with the function of AS-UMOD revealed by MDCK cells overexpressing AS-UMOD (Figure 4) and heterozygous exon 10 knockout MKTAL cells (Figure 5). The similar results obtained under conditions where the endogenous UMOD was absent (Figure 4) and present (Figures 5–7) could be explained by the lack of interaction between 2 isoforms (Figure 6A).

### AS-UMOD induction protects TAL cells in vivo and ameliorates severe IRI.

**Figure 8:**
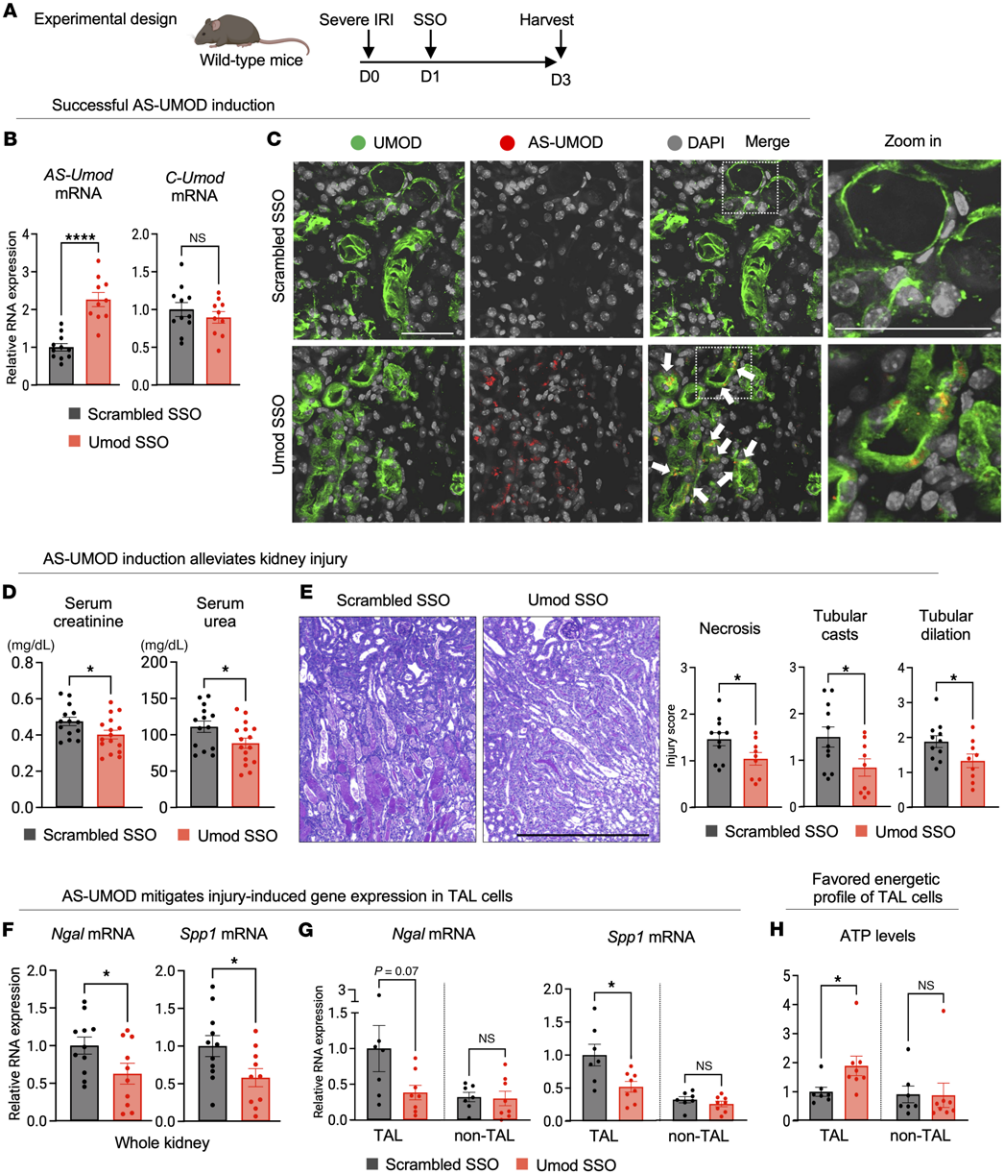
AS-UMOD induction protects TAL cells and ameliorates severe IRI. WT mice underwent severe IRI and SSO treatment (25 mg/kg) and were harvested 72 hours after IRI. (**A**) Schematic of experimental design. (**B**) Relative mRNA expression of *AS-Umod* and *C-Umod* normalized to *Gapdh*. *n* = 10–11 per group. (**C**) Immunofluorescence of murine kidneys. White arrows indicate AS-UMOD, which is induced in the cytosol of TAL cells after Umod SSO treatment. *n* = 4 mice per group. Scale bars: 50 μm. (**D**) Serum creatinine and urea concentration. *n* = 14–16 per group. (**E**) PAS-stained kidney sections and quantification of injury. *n* = 9–11 per group. Scale bar: 500 μm. (**F**) Relative mRNA expression of injury-related genes normalized to *Gapdh* in the whole kidney. *n* = 10–11 per group. (**G** and **H**) Primary TAL cells were isolated by magnetic cell separation, and cells unbound to the beads were defined as non-TAL cells. (**G**) Relative mRNA expression of injury-related genes normalized to *Gapdh* in TAL and non-TAL cells. *n* = 7–8 per group. (**H**) ATP levels normalized to protein concentration in TAL and non-TAL cells. *n* = 7–8 per group. Data were analyzed by unpaired *t* test and are represented as mean ± SEM. *P < 0.05; ****P < 0.0001.

To determine whether AS-UMOD induction during AKI is beneficial, we sought to administer Umod SSO to IRI mice (Figure 8A). We chose severe IRI mice because they failed to upregulate AS-UMOD (Figure 2A) and therefore AS-UMOD augmentation is likely to be beneficial. A challenge is that systemically administered SSOs are filtered at the glomerulus and efficiently reabsorbed by PT cells via unidentified receptors (69, 70), potentially limiting the delivery to the distal tubules. Indeed, Umod SSO injection to uninjured mice did not show significant induction (Supplemental Figure 7A). Intriguingly, however, Umod SSO significantly induced *AS-Umod* mRNA expression when administered after IRI (Figure 8B), likely due to impaired reabsorption by injured PT cells. Immunofluorescence analysis confirmed the successful induction of AS-UMOD protein in the cytoplasm of TAL cells (Figure 8C). To confirm the uptake of SSO in TAL cells, we administered ATTO 647N–conjugated Umod SSO and verified that Umod SSO was localized in the perinuclear region of TAL cells (Supplemental Figure 7B, white arrows). Note that the SSO signal was most intense in the tubular lumen (Supplemental Figure 7B, asterisks), often overlapping with casts, and not evident within LRP2-positive proximal tubular cells. These data strongly support that, after severe IRI, SSO can be delivered to distal nephron segments due to impaired reabsorption by injured PT cells.

We found that AS-UMOD augmentation mitigated AKI assessed by kidney function (Figure 8D), histological injury (Figure 8E), and injury-induced gene (*Ngal* and *Spp1*) expression (Figure 8F). We did not observe liver toxicity measured by serum alanine aminotransferase (ALT) levels both in uninjured mice (Supplemental Figure 7C) and IRI mice (Supplemental Figure 7D).

Considering the intracellular localization and function of AS-UMOD, we hypothesized that Umod SSO mitigated AKI by primarily protecting TAL cells. To test this, we isolated and analyzed primary TAL cells (53, 71) and also examined the remaining cells as a control (non-TAL cells). Successful isolation of TAL cells was confirmed by exclusive *Umod* mRNA expression in the TAL cell fraction (Supplemental Figure 7E). *Spp1* expression was reduced in TAL but not in non-TAL cells. *Ngal* expression showed a similar trend although it did not achieve statistical significance (Figure 8G). Moreover, only primary TAL cells showed higher intracellular ATP levels after Umod SSO treatment (Figure 8H), suggesting improved energy metabolism. Taken together, AS-UMOD induction improved the course of severe AKI by protecting TAL cells, likely through metabolic adaptation.

## Discussion

**Figure 9:**
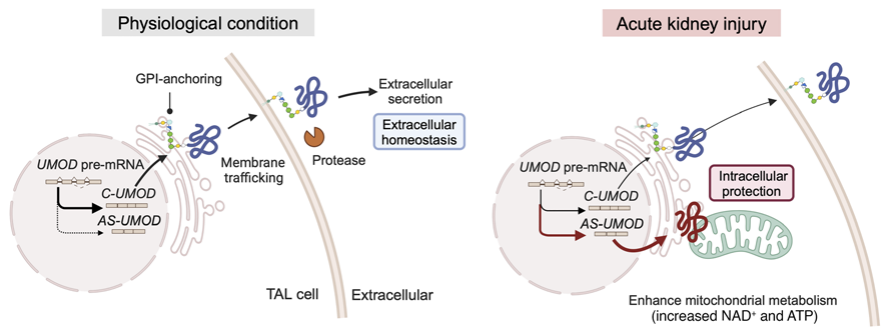
Graphical visualization of alternative splicing of UMOD. C-UMOD is a GPI-anchored protein and is sorted to the plasma membrane. C-UMOD regulates the activities of membrane transporters and maintains extracellular homeostasis once secreted into the extracellular region. AKI induces alternative splicing of UMOD and generates AS-UMOD, a non-GPI anchored isoform. AS-UMOD showed preferential localization in the mitochondria compared with C-UMOD, facilitating mitochondrial energy generation as a metabolic adaptation to cellular injury. However, mitochondrial localization of AS-UMOD remains partial, and we cannot exclude the possibility that AS-UMOD in ER could also affect mitochondrial function. The mechanism by which a portion of AS-UMOD targets the mitochondria remains unknown. The schema was created in BioRender. Nanamatsu, A. (2025) https://BioRender.com/k97g401

TAL cells maintain ATP levels (7) and are resistant to ischemic injury (6) despite their high energy demand. This requires adaptive metabolic mechanisms that have not been fully understood. The present study demonstrated that exon 10 skipping of the *UMOD* gene generates an intracellular isoform, which facilitates mitochondrial energetic adaptation during injury (visualized in Figure 9). Exon 10 skipping of the human *UMOD* gene has also been reported in the The Cancer Genome Atlas database in ExonSkipDB (Item: exon_skip_142609) (72). Our study established its presence, conservation, regulation, metabolic function, and protective role. Interestingly, UMOD S614X, an artificially generated mutant UMOD that lacks all C-terminal sequences starting from the GPI-anchoring site, became a soluble form and was secreted extracellularly (47). The difference between 2 non-GPI–anchored UMOD (AS-UMOD and UMOD S614X) is the exclusive presence of C-terminal exon 11 in AS-UMOD. Therefore, in addition to exon 10 skipping, the existence of exon 11 in AS-UMOD likely plays a role in its intracellular targeting.

We propose that increased ATP generation by AS-UMOD is important during the reperfusion phase of injury, but not hypoxia. This is supported by in vitro observation that AS-UMOD expression was induced by oxidative stress (Figure 2K), but was inhibited by hypoxia (Figure 2L). An increase in ATP production 24–72 hours after IRI, accompanied by increased mitochondrial biosynthesis markers, has been reported (73). Increasing mitochondrial metabolism, including biogenesis and NAD synthesis, is beneficial for improving the course of AKI and promoting recovery (60, 74). We propose that AS-UMOD protects TAL cells by facilitating mitochondrial metabolism and enhancing ATP generation during reperfusion injury. Of note, a certain level of physiological ROS is necessary for recovery after injury (74, 75). Considering that hydrogen peroxide induced AS-UMOD expression (Figure 2K) and AS-UMOD induction was only observed in AKI models that are linked with recovery (Figure 2, A–H), it would be interesting to hypothesize that AS-UMOD could contribute to the protection conferred to TAL cells when induction of ROS occurs during AKI. The protective effect of AS-UMOD against hypoxic treatment in transduced MDCK cells (Figure 3F) could be explained by enhanced ATP stores (76) by AS-UMOD expression.

Enhancing AS-UMOD with SSO is a potential therapeutic strategy for AKI by preserving the integrity of TAL cells through regulating UMOD expression and energy metabolism. AKI is a worldwide concern, yet therapeutic interventions are lacking. The main focus of AKI research has been PT cells, which are the most abundant cell type in the kidney (5) and are most vulnerable in experimental models of ischemic AKI (6). The importance of TAL integrity in AKI has been recently highlighted (8–12). Our results support that AS-UMOD is a TAL-protective molecule during injury, and AS-UMOD induction using Umod SSO is a unique strategy which could contribute to successful recovery. The effectiveness of post-AKI administration of SSO on ameliorating the course of injury is clinically relevant. The long-term impact of protecting TAL segments and their interactions with other neighboring cells (e.g., immune cells and fibroblasts) are the scope of future study. We found that *AS-Umod* mRNA expression remained elevated 2 weeks after Umod SSO treatment compared with scrambled SSO treatment following severe IRI (Supplemental Figure 7F), suggesting the potential for long-term intervention of Umod SSO.

Our findings expand the current understanding of alternative splicing, and therefore bear relevance to a broader cell biology context. Alternative splicing occurs in (a) cell/tissue-type-specific or (b) condition-specific manners. The cell/tissue-specific alternative splicing is a static cellular process and is typically associated with tissue identity (77). Cell type–specific alternative splicing is also linked to energy metabolism. For example, cancer cells utilize alternative splicing of pyruvate kinase to adapt to metabolic alteration (78). Condition-specific alternative splicing is a dynamic process, but its impact of on metabolic homeostasis has been underrecognized (77, 79). This study links stress-induced alternative splicing to mitochondrial metabolic adaptation. Augmentation of mitochondrial function and biogenesis is important for the recovery of several organ functions in acute and chronic disease conditions (74). Therefore, condition-specific alternative splicing might be a relevant cellular adaptation mechanism beyond the kidney. Further research will establish this paradigm as the development of transcriptome and genome sequencing technologies are now uncovering the impact and importance of alternative splicing (80).

The present study uniquely demonstrates the efficacy of SSOs in targeting distal nephron segments. Recent technological advances in nucleic acid chemistry and pharmacology have led many SSOs to be clinically approved, marking this technology as one of the most rapidly evolving therapeutic strategies (67). Despite the remarkable successes in the clinical translation, effective delivery to the target sites remains a major challenge (68). Our data suggests that SSO delivery to distal nephron segments improves after IRI, probably due to the impaired reabsorption in PT cells. ASO/SSO treatment targeting distal nephron segments may be promising for patients with AKI or CKD where the function of PT cells is impaired.

The unique association between AS-UMOD and mitochondrial transporters is reminiscent of the ability of C-UMOD (WT UMOD) to regulate the activity of the apical membrane transporters including NKCC2 (16–18). C-UMOD is proposed to serve as a scaffold for NKCC2 (16), because UMOD and NKCC2 exhibit close spatial proximity (17) and share lipid raft localization (81). It might be possible that UMOD interacts with membrane or mitochondrial transporters depending on the cellular conditions. The high expression of glutamate carriers in TAL cells (Supplemental Figure 6E) is interesting because TAL cells can utilize glutamate (82), but they do not preferentially use it under physiological conditions. Endogenous glutamate utilization might be important in the setting of injury. Increased ATP export by AS-UMOD (Figure 4K) also suggests the functional association between AS-UMOD and mitochondrial ADP/ATP carriers. However, it remains unknown whether the SLC25 carrier is required for the metabolic role of AS-UMOD and this will be the subject of future detailed studies.

The mechanism by which a portion of AS-UMOD targets the mitochondria remains unknown. Considering that AS-UMOD also interacts with ER proteins (Figure 4G) and AS-UMOD harbors an ER signal peptide at the N-terminus, there are at least 2 potential explanations: (a) AS-UMOD is initially sorted into ER and then a fraction of it is directed to mitochondria, and (b) AS-UMOD is a dual-targeted protein that can localize both in the ER and mitochondria. We cannot exclude the possibility that AS-UMOD in ER could also affect mitochondrial function by, for example, interacting with mitochondria-associated membranes (MAMs). The elucidation of the targeting/sorting mechanism of AS-UMOD in TAL cells and its relevance in ER is the subject of future studies.

In conclusion, we show that alternative splicing dynamically converts a secreted protein UMOD to an intracellular protein for metabolic adaptation of TAL cells during injury. In addition to providing a metabolic adaptation mechanism for TAL cells, this work underscores the importance of condition-specific alternative splicing in metabolic homeostasis. Enhancing alternative splicing of the *UMOD* gene might be a therapeutic intervention to improve the course of AKI.

## Methods

### Sex as a biological variable.

This study exclusively examined male mice. It is unknown whether the findings are relevant for females in 2 respects: differential UMOD expression and sensitivity to IRI. Total UMOD expression is higher in females than males both in humans and mice (43), and therefore the expression of the spliced variant will likely be different in females. In addition, females are less susceptible to AKI than males in rodents (83), and it is possible that the magnitude of AS-UMOD induction is different in males and females. For patient samples, we assessed both males and females, but the sample number was limited and was not enough to determine the difference by sex. We recognize the importance of sexual differences related to this study and plan to investigate it in the next years.

### Animal experiments.

We used 8- to 12-week-old WT male mice on a 129/SvEv background for most experiments except for those mentioned below. 129/SvEv background WT mice were purchased from Taconic Bioscience. *Umod*-knockout mice (129/ SvEv *Umod^–/–^*), (84) bred in house, were only used to validate the specificity of UMOD and AS-UMOD antibodies. We only used C57BL/6J WT mice (The Jackson Laboratory) for the LPS-induced AKI model and aristolochic acid nephropathy model. All animals were maintained in a temperature-controlled room with free access to food and water and a 12-hour day/12-hour night cycle. IRI was performed by bilateral renal pedicle clamping as described previously with 22 minutes (mild IRI) and 30 minutes clamp time (severe IRI) (25). Sham surgery was done by the same procedure without clamping. Animals were anesthetized with isoflurane. Induction occurred with 3% vapor at 1.5 l/min flow of oxygen. Animals were maintained on a rodent anesthesia circuit at 1%–2% vapor at 1.5 l/min flow of oxygen. Core temperature was maintained from 36.5–37.0°C utilizing a homeothermic warming pad. Daily saline supplementation was conducted after surgery. To establish LPS-induced AKI, 5 mg/kg LPSs from *Escherichia coli* O111:B4 (L2630, Sigma-Aldrich), dissolved in saline (50-103-1363, Fisher Scientific), was injected via intraperitoneal injection. For cisplatin-induced AKI, 20 mg/kg cisplatin (13119, Cayman Chemical), dissolved in saline, was injected via intraperitoneal injection. For the aristolochic acid nephropathy model, a single dose of aristolochic acid I (A5512, Sigma-Aldrich), dissolved in DMSO, was injected via intraperitoneal injection. Serum creatinine, urea, and ALT concentration were measured using the QuantiChrom Creatinine Assay Kit (DICT500, BioAssay Systems), QuantiChrom Urea Assay Kit (DIUR100, BioAssay Systems), and EnzyChrom Alanine Transaminase Assay Kit (EALT100, BioAssay Systems), respectively.

### Nanopore long-read RNA-Seq.

The human reference kidneys from deceased donor nephrectomies were obtained from the Indiana Donor Network. Human and mouse kidney tissues were homogenized in 800 μl of Tri Reagent using a Minilys tissue homogenizer at the highest speed for 45 seconds and then incubated for 5 minutes. Total RNA was extracted from 600 μl of the supernatant using the Direct-zol RNA Miniprep Plus (Zymo Research), including on-column DNase I digestion. The RNA was eluted in 100 μl of water and subjected to mRNA polyA enrichment using the Dynabeads mRNA DIRECT Micro Kit (61021, Thermo Fisher Scientific). After 2 rounds of washing and enrichment of mRNA following the manufacture’s protocol, polyA^+^ mRNA was eluted in 10 μl of water. Approximately 200 ng of polyA^+^ mRNA was subjected to reverse transcription and strand-switching was done using Maxima H Minus Reverse Transcriptase following the Nanopore protocol (SQK-DCS109). Following end-prep, adapter ligation, and AMPure XP bead binding, sequencing was conducted on R9.4.1 flow cells using the GridIon platform.

The basecalling was done using Guppy basecaller: guppy_basecaller --compress_fastq --fast5_out -i./fast5_pass/ -s./fastq/ --device ‘auto’ --num_callers 1 --flowcell “FLO-MIN106” --kit “SQK-DCS109”.

Mapping was carried out using Minimap2: minimap2 -a -L -t 12 -x splice --junc-bed Mus_musculus.GRCm38.101.bed --MD mm10-ont.mmi./fastq/*.fastq.gz 2>./minimap2.err >./SAM/$sample.sam.

Subsequent transcript-level analysis was performed using Bambu. To screen the differentially spliced exons in the *UMOD* gene, Sashimi plots were obtained by implementation into the Integrated Genome Viewer (IGV) browser. mRNA sequencing reads were aligned to *UMOD* gene annotations. Reads less than 1% of the total reads were excluded from the analysis.

### RT-PCR.

Human and mouse kidneys were homogenized in 1 ml of TRIzol Reagent (15596018, Thermo Fisher Scientific) using a Precellys tissue homogenizer at the highest speed for 45 seconds. Total kidney RNA was isolated following the manufacturer’s instructions. cDNA was synthesized using the High-Capacity cDNA Reverse Transcription Kit (4368814, Thermo Fisher Scientific) or ReverTra Ace (TYB-FSQ-201, Diagnocine). RT-PCR was performed using Premix Ex Taq DNA Polymerase (RR030A, Takara Bio) with 35 cycles. The sequence of PCR primers for *AS-UMOD* (exon 10 skipping *UMOD*) and *C-UMOD* (exon 10 retaining *UMOD*) in humans and in mice is as follows: human *AS-UMOD/C-UMOD* forward: GTCTACCTGCACTGTGAAGTC; human *AS-UMOD* reverse: AGACTTTCAGGAGCCCTTTC; human *C-UMOD* reverse: AAAAGCCCTTGAGACTGTGG; mouse *AS-Umod/C-Umod* forward: GTGACTCTACGAGTGAACAGTG; mouse *AS-Umod* reverse: CAGATGCTCAGGAGCCCTTG; mouse *C-Umod* reverse: AAGCAGCCTTGGACACTGAG.

We used the same forward primers for *AS-UMOD* and *C-UMOD*. The forward primers were designed to bind to *UMOD* exon 8. The reverse primers for *AS-UMOD* were designed to bind to the boundary between exons 9 and 11. Importantly, to avoid reacting within exon 9 or 11 sequence of *C-UMOD*, the *AS-UMOD* reverse primers were designed to target only a couple of nucleotides (4 for humans, 5 for mice) of the exon 9 sequence at the 3′ end of the primer, which is the crucial part for PCR reaction, while the rest 5′ side targets the exon 11 sequence. The reverse primers for *C-UMOD* were designed to target exon 10. The specificity of these primers was confirmed by qPCR using cDNA from cells which lack endogenous *UMOD* expression but overexpresses one of the isoforms.

### Sanger sequencing.

*AS-UMOD* cDNA was purified after RT-PCR using the Gene Jet PCR Gel Purification System (K0691, Thermo Fisher Scientific). Sanger sequencing was conducted by ACGT. Alignment to the reference sequence was performed using Benchling (https://benchling.com/).

### qPCR.

qPCR analysis was performed on the Thermal Cycler Dice Real Time System using THUNDERBIRD Next SYBR qPCR Mix (TYB-QPX-201, Diagnocine) or TaqMan Gene Expression Master Mix (4369016, Thermo Fisher Scientific). All the transcript levels were normalized to Gapdh mRNA levels.

The sequence of SYBR primers is as follows: mouse *AS-Umod* and *C-Umod*: the same primers used for RT-PCR; mouse *Gapdh* forward: AGCGAGACCCCACTAACATC; mouse *Gapdh* reverse: GGCGGAGATGATGACCCTTT; mouse *Hprt* forward: ACATTGTGGCCCTCTGTGTG; mouse *Hprt* reverse: TTATGTCCCCCGTTGACTGA; mouse *Glut1* forward: CAGCTGTCGGGTATCAATGC; mouse *Glut1* reverse: TCCAGCTCGCTCTACAACAA; Human *AS-UMOD* and *C-UMOD*: the same primers used for RT-PCR; human *UMOD* forward (exon 6): AAACCCATGCCACTTACAGC; human *UMOD* reverse (exon 6): CGGTCTTCAGGCTGACTTTC; human *GAPDH* forward: CAATGACCCCTTCATTGACC; human *GAPDH* reverse: TTGATTTTGGAGGGATCTCG; human *NKCC2* forward: TATGTGGTGGGATTTGCTGA; human *NKCC2* reverse: CTCCCATTCCATTCCAGCTA; dog *GRP78* forward: GGTGCCCACCAAGAAGTCTC; dog *GRP78* reverse: GGAGCAGGAGGAATTCCAGT; dog *XBP1s* forward: GAGTCCGCAGCAGGTG; dog *XBP1s* reverse: CTGTCAGAATCCATGGGG; dog *CHOP* forward: ATGGGGGTACCTGTGTTTCA; dog *CHOP* reverse: AGGTGTTCGTGACCTCTGCT; dog *TRIB3* forward: GGCACTGAGTACACCTGCAA; dog *TRIB3* reverse: GCGGGAAAAAGGTGTAGAGG; dog *GAPDH* forward: AACATCATCCCTGCTTCCAC; dog *GAPDH* reverse: GGCAGGTCAGATCCACAACT; dog *PGC1*α (*PPARGC1A*) forward: GGTCAAGATCAAGGTCCCCA; dog *PGC1*α (*PPARGC1A*) reverse: ACACAGGGGAGAATTTCGGT; dog *NRF1* forward: CAAACACGCCTTCTTCGGAA; dog *NRF1* reverse: AGACGGGGTTGGGTTTAGAG.

For Taqman qRT-PCR, we used the following Taqman probes: mouse *Umod* Mm00447649_m1 (targeting exons 2 and 3), mouse *Ngal* (*Lcn2*) Mm01324470_m1, mouse *Spp1* (*Osteopontin*) Mm00436767_m1, mouse *Kim-1* (*Havcr1*) Mm00506686_m1, mouse *Col1a1* Mm00801666_g1, mouse *Fibronectin1* (*FN1*) Mm01256744_m1, and mouse *Gapdh* (4352339E).

### Quantitative RT-PCR (qPCR) of samples from the KPMP.

Human kidney specimens collected by the KPMP consortium were acquired with informed consent and approved under a protocol by the KPMP single IRB of the University of Washington Institutional Review Board. For this analysis, we utilized available consecutive samples. We used residual RNA remaining after RNA quality index bioanalyzer analysis that is typically performed as part of the quality control for the tissue. To ensure the quality of RNA, we excluded RNA samples with an RNA integrity number of less than 2 or when leftover volume was less than 1 μl. Reverse transcription-PCR and subsequent qPCR analysis were conducted as described above. Samples falling below the detection limit due to a low original RNA concentration were excluded from analyses.

### Cell culture.

Lenti-X 293T cells (632180, Takara Bio) and MDCK cells (a gift from Kai Simons at European Molecular Biology Laboratory, Heidelberg, Germany) (85) were grown in DMEM (11965084, Thermo Fisher Scientific) supplemented with 10% FBS (A5209502, Thermo Fisher Scientific). MKTAL cells (a gift from Soline Bourgeois of University of Zurich, Zurich, Switzerland) (42) were grown in DMEM/F12 (11320033, Thermo Fisher Scientific) supplemented with 5% FBS. These cells were maintained at 37°C in a humidified 5% CO_2_ incubator. Oxidative stress was induced using hydrogen peroxide (H1009, Sigma-Aldrich). Hypoxia treatment was conducted using a hypoxia chamber (STEMCELL) with 0.1% oxygen. The serum was depleted during the hypoxia treatment. Lactate dehydrogenase (LDH) assay was performed using LDH-Glo Cytotoxicity Assay (J2380, Promega).

### Constructs, transfection (293T cells), and transduction (MDCK cells).

293T cells transiently expressing mouse UMOD were generated by transfection. pTwist CMV vectors harboring mouse C-UMOD or AS-UMOD were synthesized by Twist Bioscience. No tags were inserted. These constructs were transfected into 293T cells using Lipofectamine 2000 (11668030, Thermo Fisher Scientific). Cells were harvested 48 hours after the transfection. MDCK cells stably expressing human UMOD were generated by lentivirus transduction. pTwist ENTR vectors with human C-UMOD, AS-UMOD, and UMOD C148W sequences were generated by Twist Bioscience. No tags were inserted. The UMOD sequence was then transferred into pLenti CMV Puro DEST (17452, Addgene) using recombination reaction between attL and attR sites. These constructs were transfected into Lenti-X 293T cells with packaging vector psPAX2 (12260, Addgene) and enveloping vector pMD2.G (12259, Addgene) using Lipofectamine 2000. Lentiviral supernatant was obtained 48 hours after the transfection and was applied to MDCK cells using 6 μg/ml polybrene (sc-134220, Santa Cruz Biotechnology Inc.). Experiments were conducted below 4 passages after transduction.

### Cotransduction of C-UMOD and AS-UMOD.

pTwist Lenti SFFV Puro DEST vectors harboring human HA-C-UMOD and Myc-AS-UMOD were synthesized by Twist Bioscience. We inserted the tags after the leader peptide, between T26 and S27, based on previous reports (47, 86). Transduction to MDCK cells was conducted as described above. We established 3 MDCK cell lines: MDCK cells expressing (a) HA-C-UMOD (100%), (b) HA-C-UMOD (50%) and Myc-AS-UMOD (50%), and (c) Myc-AS-UMOD (100%). Coimmunoprecipitation was performed using the hemagglutinin tag antibody (81290-1-RR, Proteintech).

### Generation of Umod exon 10 knockout cell lines.

*Umod* exon 10 knockout MKTAL cells have been established using the CRISPR/Cas9 system, as described previously (87), with modifications. We designed 2 sgRNAs to cut the intronic region around exon 10. sgRNAs were synthesized by Synthego. The sequence of sgRNAs (including PAM) is as follows: sgRNA upstream: TGGATCGTTTGATTCGTAGGGGG; sgRNA downstream: GAGTGTGTACAATCTGCGTGAGG.

sgRNAs and Cas9 2NLS nuclease (Synthego) were transfected into MKTAL cells with SF 4D-Nucleofector X solution (V4XC-2032, Lonza) using Amaxa 4D-Nucleofector X (Lonza). Clonal isolation was performed, and genomic DNA was extracted using the Quick DNA Miniprep kit (D3025, Zymo Research). We designed PCR primers to bind around the sgRNA target sites for genotyping. The sequence for PCR primers is as follows: forward: CTTTGGTGCTTACCGTGGTT; reverse: AAGAAAAGGGCAGGGTGGAT.

Successful heterozygous knockout of *Umod* exon 10 was confirmed by PCR and subsequent Sanger sequencing of the PCR product.

### SSOs.

SSOs were designed by Integrated DNA Technologies (IDT) based on their confidential algorithms. SSOs were synthesized as 2′-O-methoxyethyl (2′-MOE) bases with phosphorothioate backbone by IDT. The sequence of SSOs is as follows: SSO (–56): TGAGGTATGTGACTTTCAAG; SSO (–36): AAGACGAGAAACATGAGAAG; SSO (–13)/Umod SSO: TGGACACCTTTGTATGAAAC; SSO (2): TGGACACTGAGGCCTGGACA; SSO (44): GTACAGAAAGAACCTAAACTTA; nontargeted SSO: GTGATCCGAGTAAGCTC; scrambled SSO (corresponds to Umod SSO): ATATGTTAGCGCCTATACGA.

MKTAL cells were treated with SSOs by reverse transfection using Lipofectamine 2000. For in vivo administration, we used HPLC-purified SSOs. Mice were administered SSOs by retro-orbital vein injection following a brief induction with isoflurane: 3% vapor at 1.5 ml/min flow of oxygen. To assess the distribution in the kidney, dye-labeled Umod SSO, where ATTO 647N-conjugated was conjugated at the 5′ end of Umod SSO, was also synthesized by IDT.

### Purification of mouse primary TAL epithelial cells.

Primary TAL cell purification was conducted by single-cell digest and subsequent magnetic cell separation as described previously (53, 71), with modifications. Mouse kidneys were decapsulated, minced, and then digested in a digestion buffer (2 mg/ml collagenase type I [SCR103, Sigma-Aldrich] and 100 U/ml DNase [79254, QIAGEN] in serum-free DMEM/F12 [Sigma-Aldrich)]) at 37°C shaker for 60 minutes. They were vortexed every 15 minutes to facilitate digestion. After single-cell digestion, DMEM/F12 with 10% FBS was added to halt the enzymatic digestion. Single-cell suspension was collected using 40 μm cell strainer (352340, Falcon). Next, Dynabeads Biotin Binder (11047, Thermo Fisher Scientific) was conjugated with mouse anti-UMOD biotinylated antibody (BAF5175, R&D Systems). After washing with 1% BSA/PBS, beads were incubated with the single-cell suspension at 4°C for 60 minutes. Both UMOD-positive cells (on-bead) and UMOD-negative cells (supernatant) were washed and collected for downstream analysis: qPCR, ATP, and protein concentration measurement. ATP levels were evaluated using CellTiter-Glo 2.0 (G9241, Promega) and normalized by protein concentration.

Further details on the methods can be found in the Supplemental Methods.

### Statistics.

Statistical analysis was performed in Excel and GraphPad Prism, version 10.4.2. No statistical methods were used to predetermine sample sizes, but our sample sizes are similar to those reported previously (26, 88). Quantitative data are presented as means ± SEM. Statistical significance was evaluated using unpaired 2-tailed *t* test (between 2 conditions) or 1-way ANOVA with embedded comparisons between 2 individual groups (among multiple conditions) with a significance level of *P* < 0.05. Blinding was performed for the animal studies (surgery, SSO injection, and harvest) and image quantification. Bubble Plot was created by SRplot (89).

### Study approval.

Animal experiments and protocols were approved by the Indiana University Animal Care and Use Committee. Analysis of the human reference kidneys from deceased donor nephrectomies obtained from the Indiana Donor Network was approved by the Indiana University Institutional Review Board (approval no.1209009674). Analysis of human biopsy kidneys from the KPMP consortium was approved by the University of Washington Institutional Review Board (approval no. 20190213).

### Data availability.

RNA-Seq data are deposited in the NCBI’s Gene Expression Omnibus database (Human kidney Nanopore PCR-free direct cDNA sequencing: NCBI GEO GSE281264; mouse kidney Nanopore PCR-free direct cDNA sequencing: NCBI GEO GSE244942 [ref. 87]). Mass spectrometry data have been uploaded to the MassIVE repository and cross referenced in ProteomeXchange (accession number MSV000094329). Data are currently password protected with user name MSV000094329_reviewer password Umod. Values for all data points in graphs are reported in the Supporting Data Values file.

## Author contributions

AN designed research studies, conducted experiments, acquired data, analyzed data, and wrote the manuscript. GJR conducted experiments. KAL edited the manuscript. RM contributed to the methodology and edited the manuscript. VL designed research studies. SK conducted experiments. DB conducted experiments and edited the manuscript. SM and AZ contributed to the methodology. YHC performed tissue processing for human samples and provided reagents. EHD and ALM conducted experiments, acquired data, analyzed data, and wrote the manuscript. MJR conducted experiments. MMK edited the manuscript. AB and CLP conducted experiments. KJK provided human samples and edited the manuscript. MTE provided human samples and edited the manuscript. JH and MK analyzed human samples. RLB designed research studies and edited the manuscript. PCD and TH designed research studies, conducted experiments, acquired data, analyzed data, and edited the manuscript. TMEA designed research studies, analyzed data, and edited the manuscript.

## Supplementary Material

Supplemental data

Unedited blot and gel images

Supporting data values

## Version 1 04/08/2025

In-Press Preview

## Version 2 06/16/2025

Electronic publication
