# Supplementary material for: Alternative splicing of uromodulin enhances mitochondrial metabolism for adaptation to stress in kidney epithelial cells
Source: J Clin Invest. 2025 Apr 8;135(12):e183343. doi: 10.1172/JCI183343 (PMC12165797; doi:10.1172/JCI183343)
Supplement: Supplemental data [file jci-135-183343-s020.pdf]

Supplementary Materials

Alternative splicing of uromodulin enhances mitochondrial metabolism for adaptation to stress in kidney epithelial cells

Authors: Azuma Nanamatsu[1*], George J. Rhodes[1], Kaice A. LaFavers[2], Radmila Micanovic[1], Virginie Lazar[1], Shehnaz Khan[1], Daria Barwinska[1], Shinichi Makino[1], Amy Zollman[1], Ying-Hua Cheng[1], Emma H Doud[3,4], Amber L. Mosley[3,4,5], Matthew J Repass[6], Malgorzata M Kamocka[1], Aravind Baride[7], Carrie L Phillips[7], Katherine J. Kelly[1,8], Michael T Eadon[1], Jonathan Himmelfarb[9], Matthias Kretzler[10], Robert L. Bacallao[1], Pierre C. Dagher[1], Takashi Hato[1,8,11], Tarek M. El-Achkar[1,2,8*].

1. Supplementary Table

2. Supplementary Figures

3. Supplementary Methods

	Reference	AKI	CKD
Sex			
Male	3	2	6
Female	6	5	6
Age	38.6 ± 7.9	41.6 ± 11.8	63.8 ± 12.8
Race			
White	8	3	9
Black or African-American	0	3	2
Asian	1	0	1
Other	0	1	0
Comorbidities			
Diabetes	0	3	11
Hypertension	0	4	10

Supplementary Table 1: Demographics of reference and disease kidney tissue specimens. Age is presented as mean ± SD.

Supplemental Figure 1

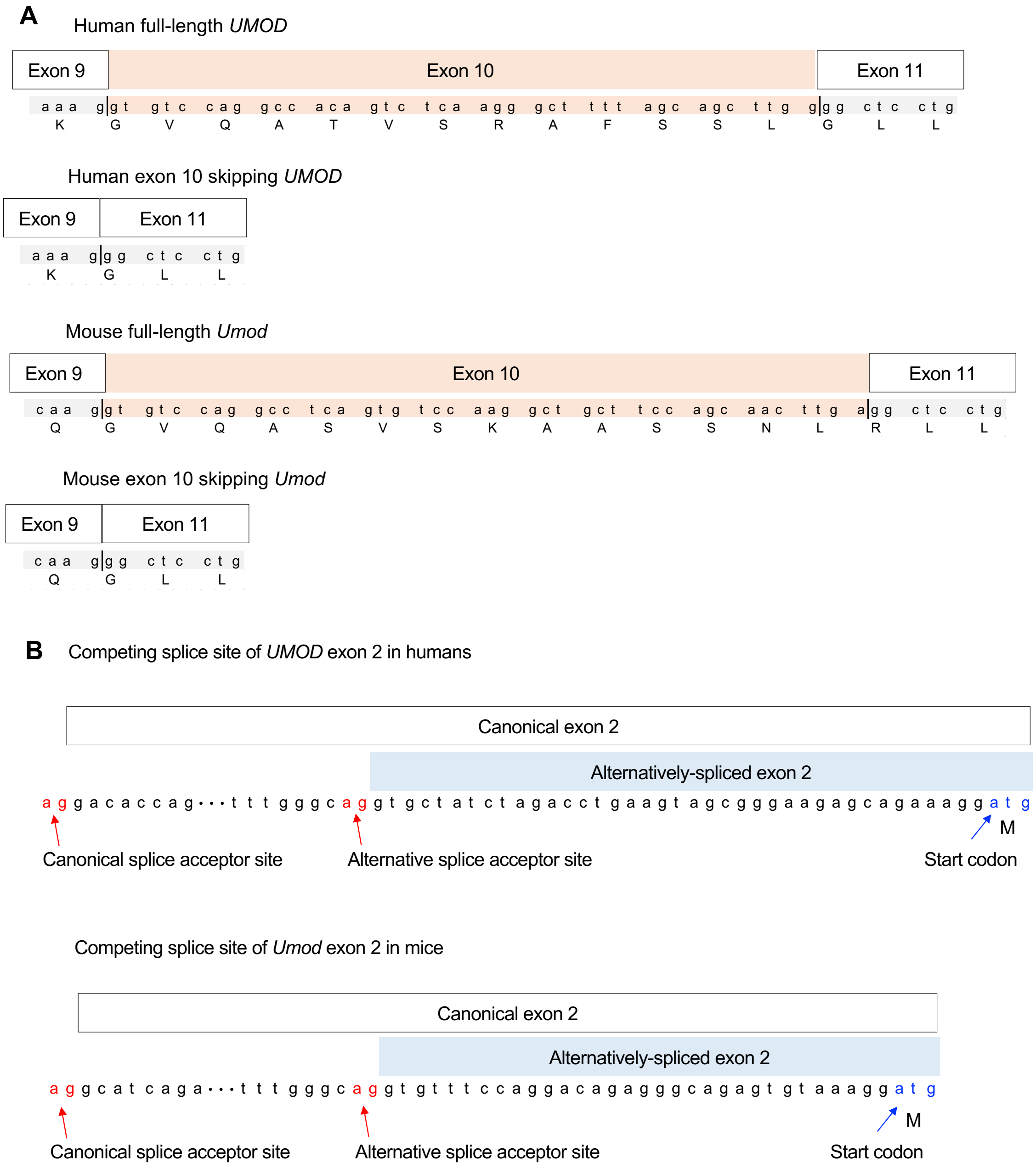

Supplementary Figure 1 (relating to Figure 1): (A) DNA and amino acid sequence of exon 10 retaining and skipping *UMOD* in humans and in mice. (B) Competing splice site of *UMOD* exon 2 in humans and in mice.

Supplemental Figure 2

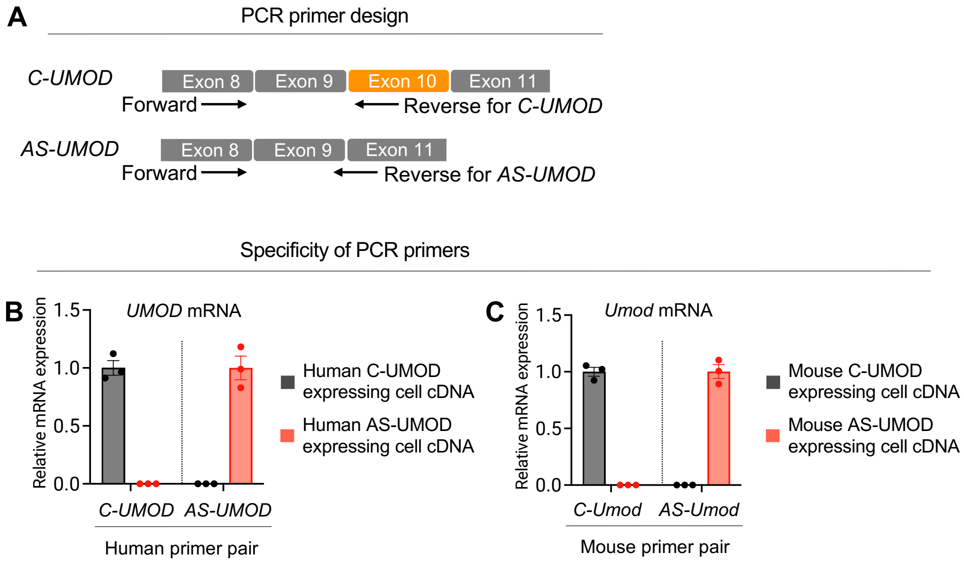

Supplementary Figure 2 (relating to Figure 1): (A) Target site of PCR primers. The same forward primers, targeting exon 8, were used for *AS-UMOD* and *C-UMOD*. The reverse primers for *C-UMOD* and *AS-UMOD* were designed to target exons 10 and the boundary between exons 9 and 11, respectively (See Methods for detail). (B) Validation of specificity of human PCR primers by qPCR using cDNA from MDCK cells which express either human C-UMOD or AS-UMOD. Human *C-UMOD* or *AS-UMOD* expression was normalized to canine *GAPDH* expression. n = 3. (C) Validation of specificity of mouse PCR primers by qPCR using cDNA from 293T cells which express either mouse C-UMOD or AS-UMOD. Mouse *C-Umod* or AS-*Umod* expression was normalized to human *GAPDH* expression. n = 3. Values are presented as mean ± SEM.

Supplemental Figure 3

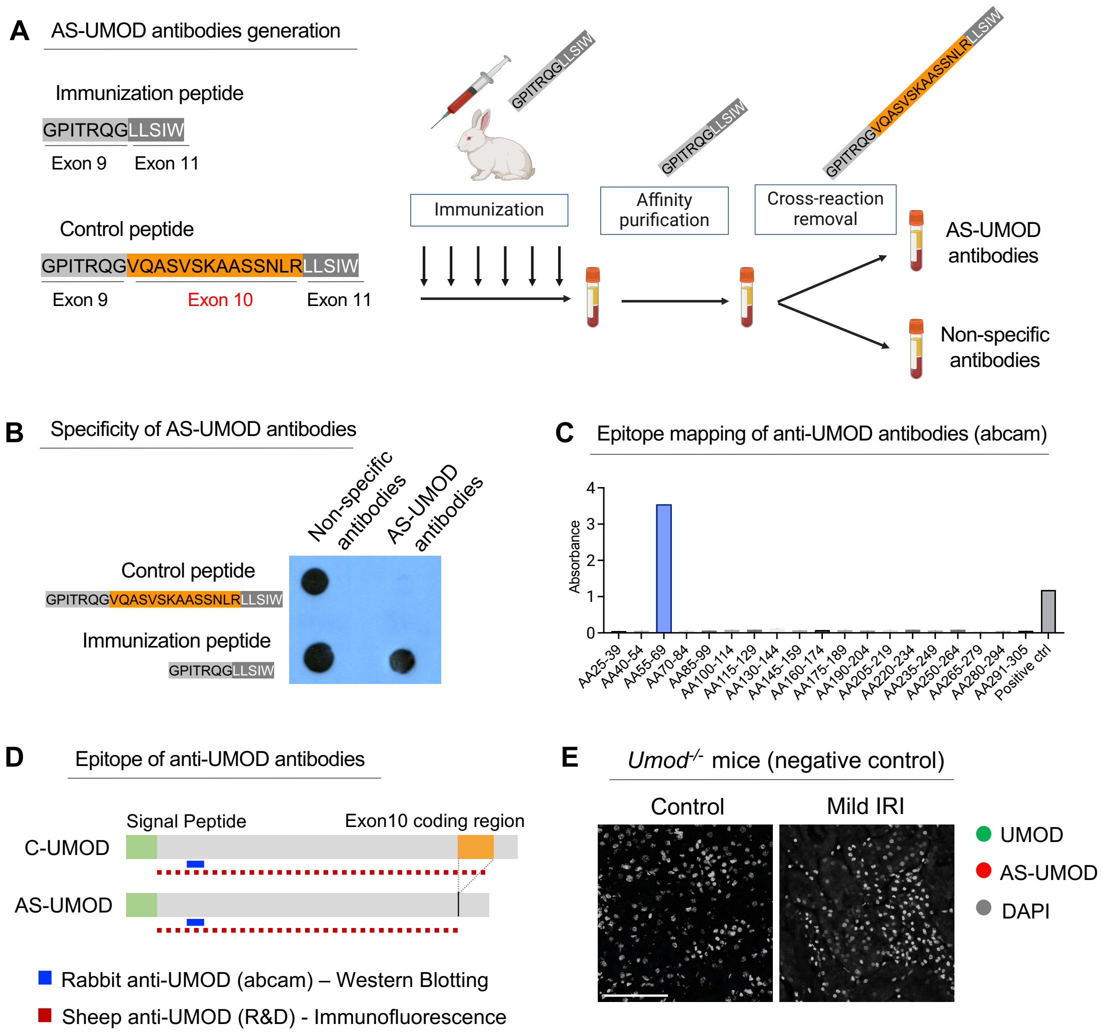

Supplementary Figure 3 (relating to Figure 2): (A) Schematic of AS-UMOD antibodies generation. Immunization peptide corresponds to the boundary of mouse UMOD exons 9 and 11 coding amino acid sequence. Immunization and affinity purification were performed using this peptide. To exclude the antibodies that cross-react with C-UMOD, counter-screening was performed by repeating affinity purification using control peptide that spans the exon 9, 10 and 11 coding amino acid sequence. The schema was created in BioRender. Nanamatsu, A. (2025) https://BioRender.com/z3t1dit (B) Dot blot verified the specificity of AS-UMOD antibodies. (C) Epitope mapping of anti-UMOD antibodies (Abcam) identified that their epitope is at the N-terminal side (AA 55-69). A combination of UMOD peptide (AA 371–385) and mouse anti-UMOD antibodies (sc-271022, Santa Cruz Biotechnology) was used as a positive control. (D) Visualization of the epitope of anti-UMOD antibodies used in this study. (E) Immunofluorescence of subcortical region of *Umod*$^{-/-}$ murine kidneys 24 hours after IRI as a negative control for anti-UMOD and anti-AS-UMOD antibodies. n=3. Bars = 100 μm.

Supplemental Figure 4

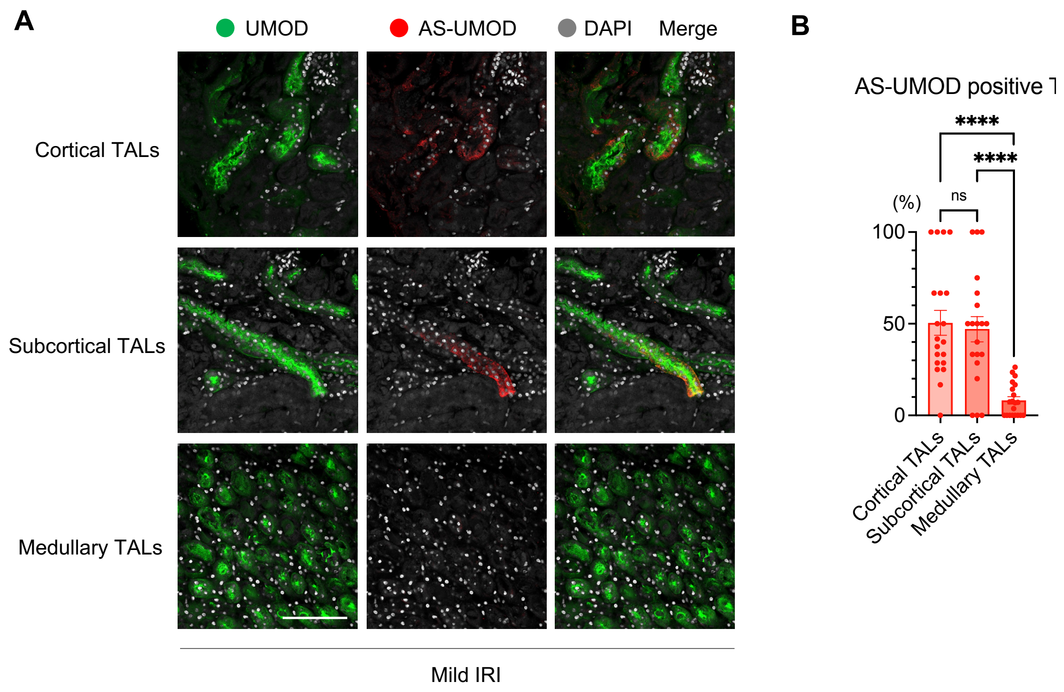

Supplementary Figure 4 (relating to Figure 2): (A) Immunofluorescence of murine kidneys 24 hours after mild IRI in different TAL segments (UMOD positive tubules). n=5 mice per group. Bars = 100 µm. (B) Ratio of AS-UMOD positive TALs in each TAL segment. n = 20 images for each segment from 5 kidneys. Data were analyzed by one-way ANOVA with embedded comparisons between two individual groups and were presented as mean ± SEM. ****$p<0.0001$, ns: not significant.

Supplemental Figure 5

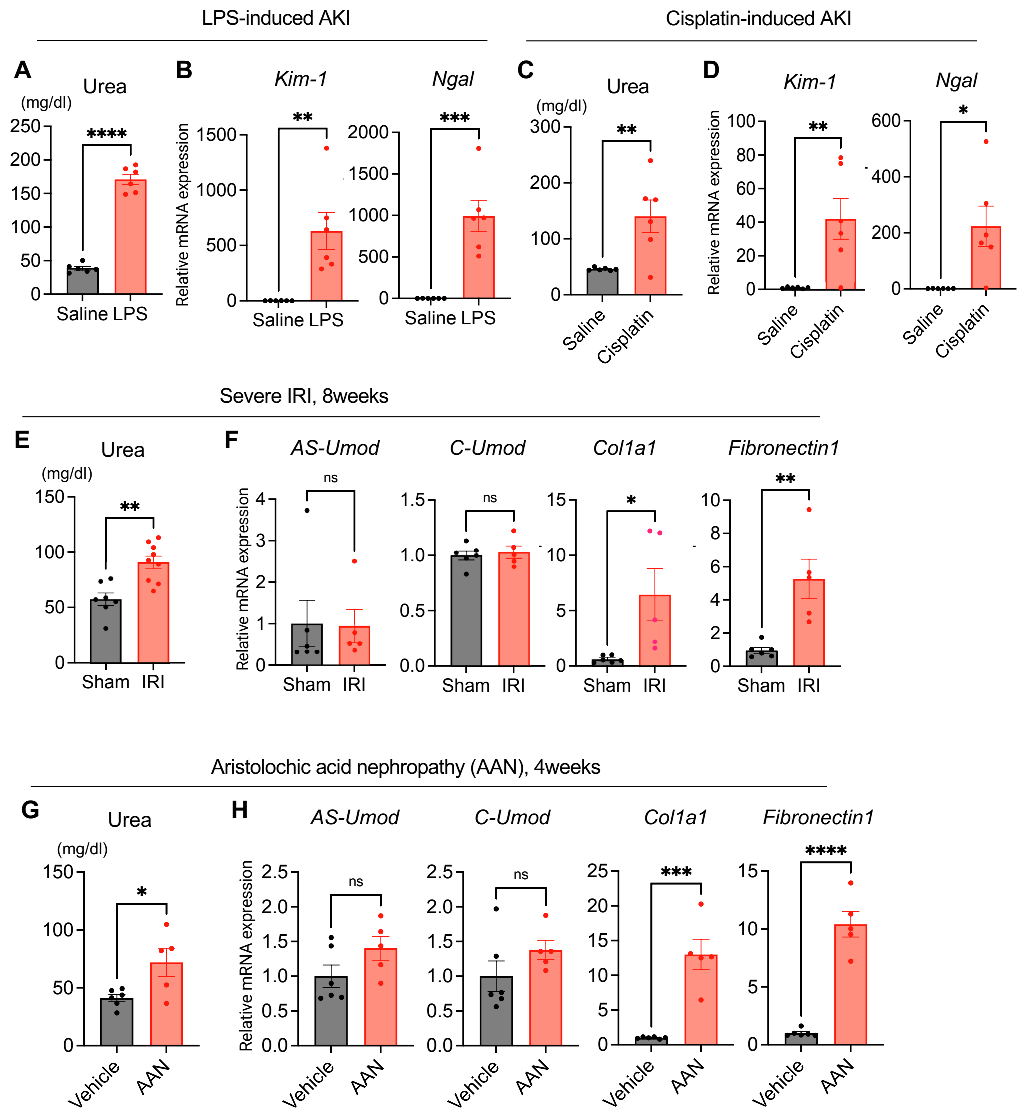

Supplementary Figure 5 (relating to Figure 2): (A) Serum urea concentration 24 hours after 5mg/kg LPS injection. n=6 per group. (B) Relative mRNA expression of *AS-Umod* and *C-Umod* normalized to *Gapdh* 24 hours after 5mg/kg LPS injection. n=6 per group. (C) Serum urea concentration 72 hours after 20mg/kg cisplatin injection. n=6 per group. (D) Relative mRNA expression of *AS-Umod* and *C-Umod* normalized to *Gapdh* 72 hours after 20mg/kg cisplatin injection. n=6 per group. (E) Serum urea concentration in severe IRI mice at 8-week timepoint. n = 7-9 per group. (F) Relative mRNA expression of *AS-Umod, C-Umod, Col1a1, Fibronectin1* normalized to *Gapdh* in severe IRI mice at 8-week timepoint. n = 5-6 per group. (G) Serum urea concentration 4 weeks after a single dose injection of aristolochic acid. n = 5-6 per group. AAN, aristolochic acid nephropathy. (H) Relative mRNA expression of *AS-Umod, C-Umod, Col1a1, Fibronectin1* normalized to *Gapdh* 4 weeks after a single dose injection of aristolochic acid. n = 5-6 per group. Data were analyzed by unpaired t test and were presented as mean ± SEM. *p<0.05, **p<0.01, ***p<0.001, ****p<0.0001, ns: not significant.

Supplemental Figure 6

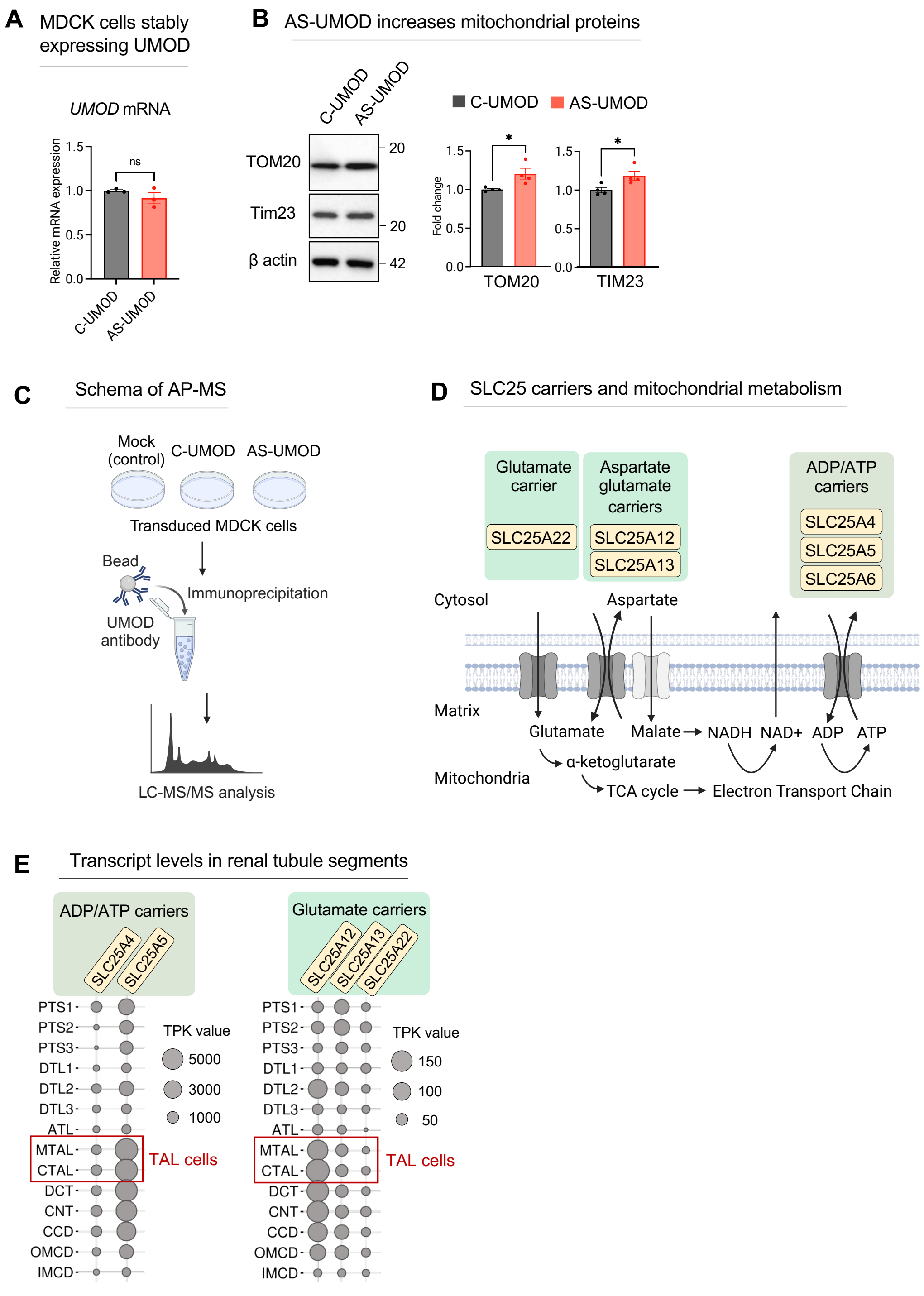

Supplementary Figure 6 (relating to Figures 3 and 4): (A) Relative mRNA expression of *UMOD* normalized to *GAPDH* in MDCK cells expressing C-UMOD or AS-UMOD. n=3. (B) Immunoblotting and densitometric analysis of mitochondrial proteins in MDCK cells expressing C-UMOD or AS-UMOD. n = 4. (C) Schema of affinity-purification coupled to mass spectrometry (AP-MS). MS was performed following anti-UMOD immunoprecipitation of MDCK cells expressing mock, C-UMOD or AS-UMOD. Created in BioRender. Nanamatsu, A. (2025) https://BioRender.com/1wi34cl (D) Schema showing how SLC25 carriers regulate mitochondrial metabolism. Created in BioRender. Nanamatsu, A. (2025) https://BioRender.com/9uz1s66 (E) Transcript levels of SLC25 carriers in renal tubule segments obtained from available RNA sequencing data of mouse micro-dissected segments (Chen et al, 2021). PTS1, the initial segment of the proximal tubule; PTS2, proximal straight tubule in cortical medullary rays; PTS3, last segment of the proximal straight tubule in the outer stripe of outer medulla; DTL1, the short descending limb of the loop of Henle; DTL2, long descending limb of the loop of Henle in the outer medulla; DTL3, long descending limb of the loop of Henle in the inner medulla; ATL, thin ascending limb of the loop of Henle; MTAL, medullary thick ascending limb of the loop of Henle; CTAL, cortical thick ascending limb of the loop of Henle; MD, mecula densa; DCT, distal convoluted tubule; CNT, connecting tubule; CCD, cortical collecting duct; OMCD, outer medullary collecting duct; IMCD, inner medullary collecting duct. Data were analyzed by unpaired t test and were presented as mean ± SEM. *p<0.05, ns: not significant.

Supplemental Figure 7

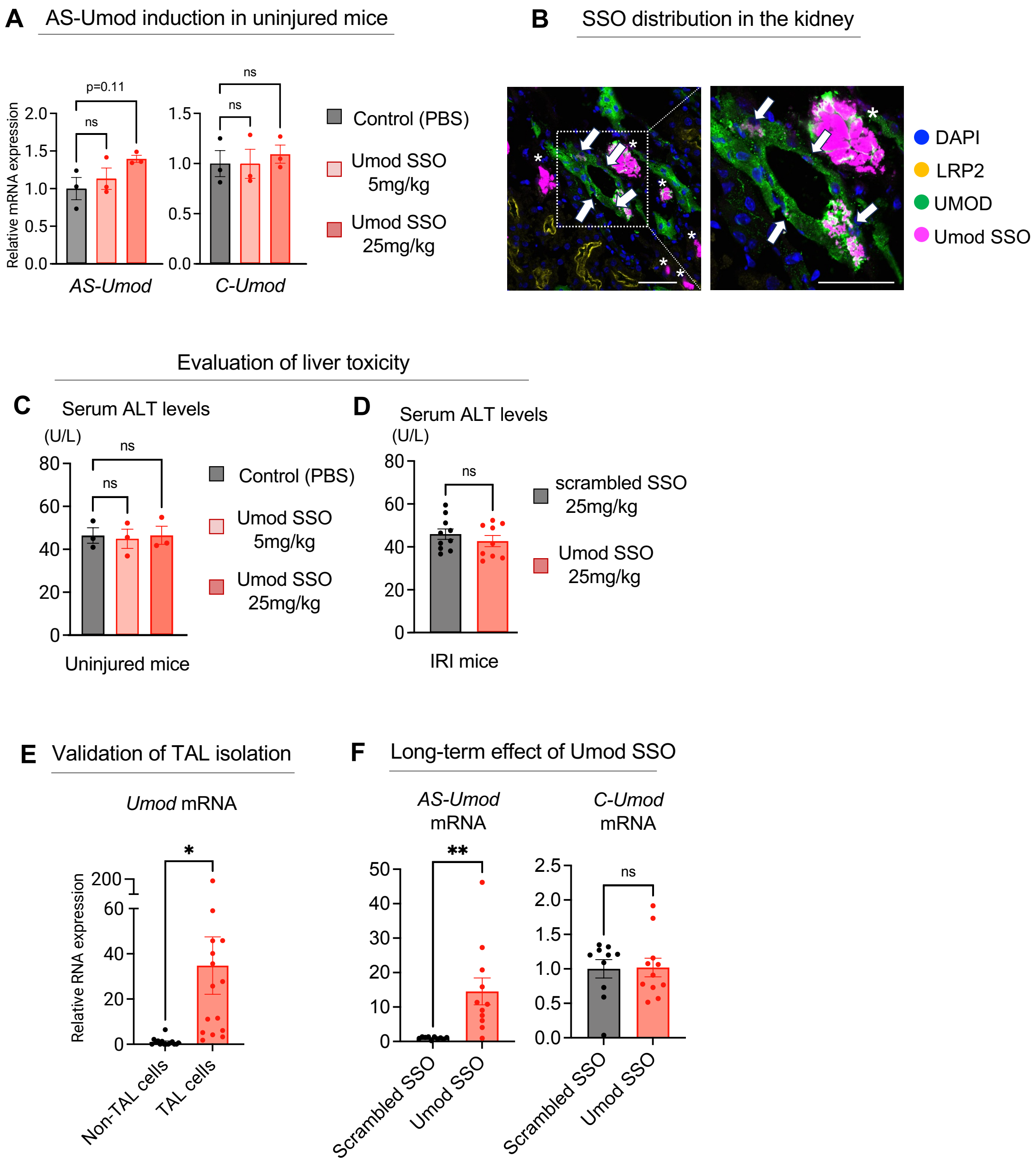

Supplementary Figure 7 (relating to Figure 8): (A) Relative mRNA expression of *AS-Umod* and *C-Umod* normalized to *Gapdh* after SSO treatment without IRI. Kidney mRNA was isolated 48 hours after PBS (control) or Umod SSO treatment. n = 3 per group. (B) Immunofluorescence of subcortical region of murine kidneys treated with ATTO 647N-labelled Umod SSO after severe IRI. Murine kidneys were harvested 24 hours after the SSO injection. White arrows indicate Umod SSO which is localized in the perinuclear region of TAL cells. Asterisk shows SSO in the lumen; some were associated tubular casts. Scale Bar = 50 µm. n=3 mice. (C) Serum ALT concentration 48 hours after PBS (control) or Umod SSO treatment. n = 3 per group. ALT = alanine aminotransferase. (D) Serum ALT concentration after severe IRI with SSO treatment. n = 9-10 per group. (E) Relative mRNA expression of AS-Umod normalized to Gapdh in non-TAL and TAL cells. n = 15 per group. (F) Relative mRNA expression of *AS-Umod* and *C-Umod* normalized to *Gapdh* 2 weeks after severe IRI and SSO treatment. n=10 or 11 per group. Data were analyzed by unpaired t test (between two conditions, D-F) or one-way ANOVA with embedded comparisons between two individual groups (among multiple conditions, A and C) and were presented as mean ± SEM. *p<0.05, **p<0.01, ns: not significant.

Supplementary Methods

Immunoblotting

MDCK and MKTAL cells were lysed using RIPA Lysis and Extraction Buffer (89900, Thermo Fisher Scientific) with Halt Protease and Phosphatase Inhibitor Single-Use Cocktail (78442, Thermo Fisher Scientific). The lysates were centrifuged at 14000*g* for 10 minutes at 4°C, and then the supernatants were collected. Media from MDCK and MKTAL cells were concentrated using Nanosep centrifugal filters with 10 kDa cutoff filters (OD010C34, Pall Corporation). Integrale, Tokushima, Japan). The lysates and media were then denatured for 20 minutes at 60°C using 4X Bolt LDS Sample Buffer (B0007, Thermo Fisher Scientific) with 200mM DTT (700416-1, Cayman Chemical). Total protein concentration was determined using DC (detergent compatible) Protein Assay (5000113-5000115, BioRad). For immunoblotting, the following primary antibodies were used: rabbit anti-UMOD (1:5000, ab207170, Abcam), rabbit anti-β-actin (1:2000, 20536-1-AP, Proteintech), rabbit anti-VDAC1 (1:2000, 55259-1-AP, Proteintech), rabbit anti-α-tubulin (1:2000, 11224-1-AP, Proteintech), NaKATPase (1:2000, sc-28800, Santa Cruz Biotechnology), rabbit anti-TOM20 (1:5000, 11802-1-AP, Proteintech), mouse anti-Tim23 (1:2000, 611222, BD Transduction), rabbit anti-SLC25A4 (1:2000, NBP2-92642, Novus Biologicals), rabbit anti-SLC25A5 (1:2000, 14671, Cell Signaling), rabbit anti-SLC25A6 (1:2000, 14841-1-AP, Proteintech), rabbit anti-SLC25A12 (1:2000, ab200201, Abcam), rabbit anti-SLC25A22 (1:2000, 25402-1-AP, Proteintech), mouse anti-HA (1:2000, 2367, Cell Signaling Technology), mouse anti-Myc (1:2000, 2276, Cell Signaling Technology) and mouse anti-CANX (1:2000, C4731, Sigma-Aldrich). HRP-conjugated anti-IgG antibodies (Proteintech) were used as

secondary antibodies. Loading controls (β-actin for cell lysates and Coomassie brilliant blue staining for media) were run on the same membrane as the target protein after stripping using Restore Western Blot Stripping Buffer (21059, Thermo Fisher Scientific). Coomassie brilliant blue staining was conducted using One-Step Blue® Protein Gel Stain (21003-1L, Biotium). The signals were quantified by ImageJ.

Immunostaining

Mouse kidneys were fixed with 4% paraformaldehyde and cut into 50 μm sections using vibratome (Leica). MDCK and MKTAL cells were fixed with 4% paraformaldehyde and permeabilized with 0.1% Triton X/PBS. To stain mitochondria, MDCK cells were treated with 100nM MitoTracker Deep Red FM (M22426, Thermo Fisher Scientific) for 45 minutes before fixation. Mouse kidney sections and MDCK cells were incubated with the following primary antibodies: sheep anti-UMOD (1:300, AF5175, R&D Systems), rabbit anti-AS-UMOD antibodies and rabbit anti-LRP2 (ab76969, Abcam). AlexaFluor 488 and 647 were used as the secondary antibodies. Can Get Signal immunostain (TYB-NKB-401, Diagnocine) was used to enhance antigen-antibody reactions. The stained sections were mounted with ProLong Glass (P36982, Thermo Fisher Scientific). Immunofluorescence images were obtained using SP8 Confocal Microscope (Leica). Quantification of apical membrane localization of UMOD in mouse kidneys was performed as described previously(1) with modifications. Apical membrane signal intensity of UMOD was determined using the Brush tool and ROI manager in image J with an appropriate border in a 3 pixel. The signal intensity for whole tubules excluding nucleus were quantified using freehand selections in image J and compared to the

mean signal intensity of the entire cell excluding the nucleus. Colocalization between UMOD and mitochondria was determined by Manders colocalization tests as described previously(2–4). We used Coloc2 Plugins from Fiji(5) to conduct Manders colocalization tests.

AS-UMOD antibody generation

Rabbits were immunized by a synthetic peptide antigen (Cys-GPITRQGLLSIW) which corresponds to the amino acid coding sequence at the boundary of mouse UMOD exons 9 and 11. After the 6th immunization, serum was collected and affinity purification was conducted using the peptide antigen. To remove antibodies which react with C-UMOD, a cross-reaction removal step was performed by affinity purification using a control peptide (Cys-GPITRQGVQASVSKAASSNLRLLSIW) which corresponds to the amino acid sequence across exon 9, 10 and 11 coding region. The specificity was validated by dot blot using these antibodies (1:3000) and 7ng peptide antigen or control peptide.

Epitope Mapping of rabbit anti-UMOD monoclonal antibody

The antigen of rabbit anti-UMOD monoclonal antibody (ab207170, abcam) was human UMOD protein antigen (AA25-305) based on the manufacturer's information. To verify and define the exact epitope, we used an epitope mapping approach(6). A peptide library was designed and generated by WatsonBio Sciences. 18 peptides, each 15 amino acids in length, were used to cover the human UMOD sequence AA25-305. Epitope mapping of rabbit anti-UMOD monoclonal antibody was performed in a direct

ELISA. A combination of UMOD peptide (AA 371–385) and mouse anti-UMOD monoclonal antibody (sc-271022, Santa Cruz Biotechnology) was used as a positive control based on our previous report(6).

Mitochondrial isolation

Mitochondrial isolation and cellular fractionation and were performed by differential centrifugation, as described previously(7, 8). Cells in 10cm dishes were incubated with PBS supplemented with 5mM EDTA for 1 minute, scraped and collected in a 15ml conical tube. After a brief centrifugation, the cell pellet was washed with PBS and resuspended in mitochondrial isolation buffer (250 mM sucrose, 1 mM EDTA, 50mM Tris-HCl and Halt Protease and Phosphatase Inhibitor Single-Use Cocktail). The pellet was homogenized and centrifuged at 600*g* for 10 min at 4°C to remove cell debris. The pellet was discarded. A portion of the supernatant was collected as whole cell lysate and lysed with RIPA Lysis and Extraction Buffer. The remaining supernatant was transferred to another tube, and centrifuged at 7000*g* for 10 min at 4°C. The pellet was collected as mitochondrial fraction. The supernatant was transferred to another tube, and centrifuged at 20000*g* for 10 min at 4°C. The supernatant and the pellet were collected as cytosol fraction and membrane fraction, respectively. The mitochondrial fraction was resuspended with mitochondrial isolation buffer and centrifuged at 10000*g* for 10 min at 4°C and the pellet was then lysed with RIPA Lysis and Extraction Buffer. ATP/ADP ratio was measured using EnzyLightTM ADP Assay Kit (EADP-100, BioAssay Systems). Glutamate levels were evaluated by Glutamate-Glo Assay (J7021, Promega).

Mitochondrial respiration measurement

Mitochondrial oxygen consumption rates were measured by Mito Stress Test using Seahorse XFp (Agilent Technologies). MDCK cells were seeded in Seahorse XFp miniplates overnight. MDCK cells were then maintained in Seahorse XF assay media in a CO2-free incubator at 37 °C for 45 minutes. Seahorse XF assay media was supplemented with 25mM D-Glucose ad 4mM L-Glutamine, and pH was adjusted to 7.4. For Mito Stress Test, we used 1 μM oligomycin, 1 μM carbonyl cyanide *p*-trifluoromethoxyphenylhydrazone (FCCP) and 0.5 μM Antimycin A/Rotenone. FCCP concentration was optimized by a preliminary titration experiment.

ADP/ATP carrier activity measurement

ATP transport activity by ADP/ATP carriers was measured as described previously (9) with modifications. 10 μg freshly isolated mitochondria were incubated with ADP reaction buffer (250 mM sucrose, 20 mM HEPES, 10 mM KCl, 3 mM KH2PO4, 1 mM EGTA, 1 mM MgCl2, 1 mM malate and 1 mM pyruvate, pH 7.6) and RealTime-Glo Extracellular ATP Assay Substrate (GA5010, Promega) in white/clear bottom 96-well plates. After 10 min, external 25 μM ADP (A2754, Sigma-Aldrich) was added. Exported ATP levels were measured by luminescence signal. The initial linear part of the reaction curve was used to assess exported ATP. External ATP levels of mitochondria without ADP addition served as background control.

Transmission electron microscopy

MDCK cells stably expressing UMOD grown on 6cm dishes were washed twice with 0.2M sodium cacodylate (50-980-2310, Fisher Scientific) and then fixed with 2.5% glutaraldehyde in sodium cacodylate (50-259-41, Fisher Scientific) for 1 hour at room temperature. The cells were transferred into a 2 mL centrifuge tube by scraping from the petri dish and then centrifuged at 5000 rpm for 3 minutes to form a pellet. A tissue processing protocol was performed using microwave (PELCO BioWave Pro, Tedpella Inc.) to fix and embed the tissue in resin. In brief, the specimen was first subjected to microwave at 150W power and then rinsed with cacodylate buffer. The specimen was fixed with 2% Osmium tetroxide at 100 W power and rinsed with cacodylate buffer. The specimen was dehydrated in a sequence with 75% ethanol, 100% ethanol (3-cycles), and propylene oxide. The preliminary resin infiltration was performed with 1:1 ratio of propylene oxide and resin at 350 W power. The resin was prepared by mixing 1:1 ratio of SPURR Low Viscosity Embed Kit and EMbed 812 Embedding Kit (Electron Microscopy Supplies). The final resin infiltration was performed by embedding the resin into the specimen at 350 W power. The tissue embedded in the resin transferred into a mold and incubated at 55 °C for polymerization. Thin sections were cut and stained with 1% UranyLess (Lanthanide acetate, Electron Microscopy Supplies) and 1% Lead citrate (Electron Microscopy Supplies). Electron micrographs were captured with Hitachi 7800 and FEI Tecnai G2 Spirit electron microscopes with 80K acceleration voltage. The Images were analyzed using ImageJ.

Affinity purification coupled to mass spectrometry (AP-MS)

Sample preparation, mass spectrometry analysis, bioinformatics, and data evaluation were performed in collaboration with the Indiana University Proteomics Center for Proteome Analysis at the Indiana University School of Medicine (IUSM) similarly to previously published protocols(10). MDCK cells cultured in 10cm dishes were lysed using Pierce IP Lysis Buffer (87787, Thermo Fisher Scientific) with Halt Protease and Phosphatase Inhibitor Single-Use Cocktail. The lysates were centrifuged at 14000*g* for 10 minutes at 4°C, and the supernatants were collected. A portion of the supernatant was kept as an input sample. The remaining supernatant was incubated with sheep anti-UMOD antibody (AF5144, R&D Systems) for overnight at 4°C, and then incubated with Pierce Protein A/G Agarose (20421, Thermo Fisher Scientific) for 4 hours at 4°C. Beads were washed with TBS. 1/4th of the beads were denatured for immunoblotting and the remaining beads were submitted to the IUSM Center for proteome analysis.

Sample Preparation for AP-MS

On bead proteins were denatured in 8 M urea, 100 mM Tris-HCl, pH 8.5 and reduced with 5 mM tris(2-carboxyethyl)phosphine hydrochloride (TCEP, C4706, Sigma-Aldrich) for 30 minutes at room temperature. Samples were then alkylated with 10 mM chloroacetamide (CAA, C0267, Sigma Aldrich) for 30 min at room temperature in the dark, prior to dilution with 50 mM Tris.HCl, pH 8.5 to a final urea concentration of 2 M for Trypsin/Lys-C based overnight protein digestion at 37 ºC (0.5 µg protease, Mass Spectrometry grade, V5072, Promega)

Peptide Purification and Labeling for AP-MS

Digestions were acidified with trifluoroacetic acid (TFA, 0.5% v/v) and desalted on Pierce C18 spin columns (89870, Thermo Fisher Scientific) with a wash of 0.5% TFA followed by elution in 70% acetonitrile 0.1% formic acid (FA).

Nano-LC-MS/MS

Mass spectrometry was performed utilizing an EASY-nLC 1200 HPLC system (014993, Thermo Fisher Scientific) coupled to Exploris 480™ mass spectrometer with FAIMSpro interface (Thermo Fisher Scientific). 1/5th of each fraction was loaded onto a 25 cm EasySpray column (ES902, Thermo Fisher Scientific) at 350 nL/min. The gradient was held at 5% B for 5 minutes (Mobile phases A: 0.1% formic acid (FA), water; B: 0.1% FA, 80% Acetonitrile (LS122500, Thermo Fisher Scientific), then increased from 4-30%B over 98 minutes; 30-80% B over 10 mins; held at 80% for 2 minutes; and dropping from 80-4% B over the final 5 min. The mass spectrometer was operated in positive ion mode, default charge state of 2, advanced peak determination on, and lock mass of 445.12003. Three FAIMS CVs were utilized (-40 CV; -55 CV; -70CV) each with a cycle time of 1.3 s and with identical MS and MS2 parameters. Precursor scans (m/z 375-1500) were done with an orbitrap resolution of 120000, RF lens% 40, automatic maximum inject time, standard AGC target, minimum MS2 intensity threshold of 5e3, MIPS mode to peptide, including charges of 2 to 7 for fragmentation with 30 sec dynamic exclusion. MS2 scans were performed with a quadrupole isolation window of 1.6 m/z, 30% HCD CE, 15000 resolution, standard AGC target, automatic maximum IT, fixed first mass of 110 m/z.

AP-MS Data Analysis

Resulting RAW files were analyzed in Proteome Discover™ 2.5 (Thermo Fisher Scientific) with *H. sapiens* UMOD sequence plus *C. lupus familiaris* reference proteome FASTA (downloaded from Uniprot 051823 with 59100 entries) plus common contaminants (73 entries(11)). SEQUEST HT searches were conducted with a maximum number of 3 missed cleavages; precursor mass tolerance of 10 ppm, and a fragment mass tolerance of 0.02 Da. Static modifications used for the search were carbamidomethylation on cysteine (C). Dynamic modifications included and oxidation of methionine (M), deamidation of asparagine or arginine, phosphorylation on serine, threonine or tyrosine, and acetylation, methionine loss, or methionine loss plus acetylation on protein N-termini. Percolator False Discovery Rate was set to a strict peptide spectral match FDR setting of 0.01 and a relaxed setting of 0.05. Results were loaded into Scaffold Q+S 5.2.2 (Proteome Software) for viewing. The interacting molecules of C-UMOD and AS-UMOD were determined by comparison with the results of mock (negative control) cells. We defined interacting molecules as having greater than fourfold change between isoform and mock expressing cells(12), and $p < 0.005$ (Fisher's Exact Test).

NAD^+ measurement

NAD^+ levels in MDCK and MKTAL cells were measured using NAD/NADH Assay Kit (Colorimetric) (ab65348, Abcam). NAD^+ concentration was normalized to protein concentration.

Histology

Kidney tissues were collected and fixed in 4% PFA and embedded in paraffin. Paraffin embedding, tissue sectioning and PAS staining were performed by Histology Lab Service Core in Indiana University. Brightfield images were taken by Keyence BZ-810. Quantification of injury (necrosis, tubular dilation and tubular casts) was performed as described previously(13, 14).Ten random fields from each kidney were selected, and injury scores from 0 to 5 were evaluated based on the percentage of injured tubules: (0) 0 – 4%, (1) 5–24%, (2) 25– 49%, (3) 50 –74%, (4) 75–99%, (5) 100%.

References

1. LaFavers KA, et al. The kidney protects against sepsis by producing systemic uromodulin. *Am J Physiol Renal Physiol*. 2022;323(2):F212–F226.
2. Dunn KW, Kamocka MM, McDonald JH. A practical guide to evaluating colocalization in biological microscopy. *American Journal of Physiology-Cell Physiology*. 2011;300(4):C723–C742.
3. Naón D, et al. Splice variants of mitofusin 2 shape the endoplasmic reticulum and tether it to mitochondria. *Science*. 2023;380(6651):eadh9351.
4. Olinger E, et al. An intermediate-effect size variant in UMOD confers risk for chronic kidney disease. *Proc Natl Acad Sci U S A*. 2022;119(33):e2114734119.
5. Schindelin J, et al. Fiji: an open-source platform for biological-image analysis. *Nat Methods*. 2012;9(7):676–682.
6. Micanovic R, et al. The kidney releases a nonpolymerizing form of uromodulin in the urine and circulation that retains the external hydrophobic patch domain. *Am J Physiol Renal Physiol*. 2022;322(4):F403–F418.
7. Lampl T, et al. Isolation and functional analysis of mitochondria from cultured cells and mouse tissue. *J Vis Exp*. 2015;(97):52076.
8. Adriaenssens E, et al. Small heat shock proteins operate as molecular chaperones in the mitochondrial intermembrane space. *Nat Cell Biol*. 2023;25(3):467–480.
9. Hoogstraten CA, et al. Metabolic impact of genetic and chemical ADP/ATP carrier inhibition in renal proximal tubule epithelial cells. *Arch Toxicol*. 2023;97(7):1927–1941.
10. Baker KM, et al. Mapping the Anti-Cancer Activity of α-Connexin Carboxyl-Terminal (aCT1) Peptide in Resistant HER2+ Breast Cancer. *Cancers (Basel)*. 2024;16(2):423.
11. Orsburn BC. Proteome Discoverer-A Community Enhanced Data Processing Suite for Protein Informatics. *Proteomes*. 2021;9(1):15.
12. Chou C-L, et al. Identification of UT-A1- and AQP2-interacting proteins in rat inner medullary collecting duct. *Am J Physiol Cell Physiol*. 2018;314(1):C99–C117.
13. Micanovic R, et al. Tamm-Horsfall Protein Regulates Mononuclear Phagocytes in the Kidney. *J Am Soc Nephrol*. 2018;29(3):841–856.
14. El-Achkar TM, et al. Tamm-Horsfall protein protects the kidney from ischemic injury by decreasing inflammation and altering TLR4 expression. *Am J Physiol Renal Physiol*. 2008;295(2):F534-544.
