# Supplementary material for: Alternative splicing of uromodulin enhances mitochondrial metabolism for adaptation to stress in kidney epithelial cells
Source: J Clin Invest. 2025 Apr 8;135(12):e183343. doi: 10.1172/JCI183343 (PMC12165797; doi:10.1172/JCI183343)

Figure 1F

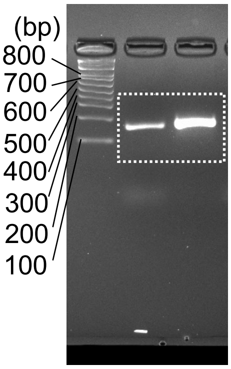

Figure 1H

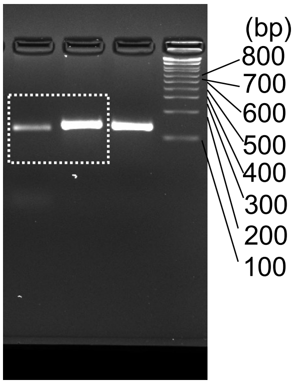

Figure 3A

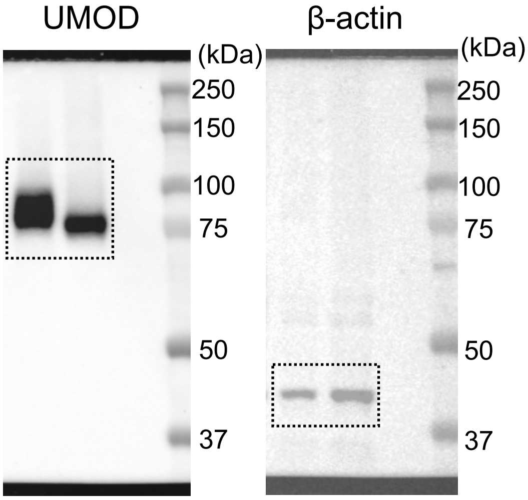

Figure 3B

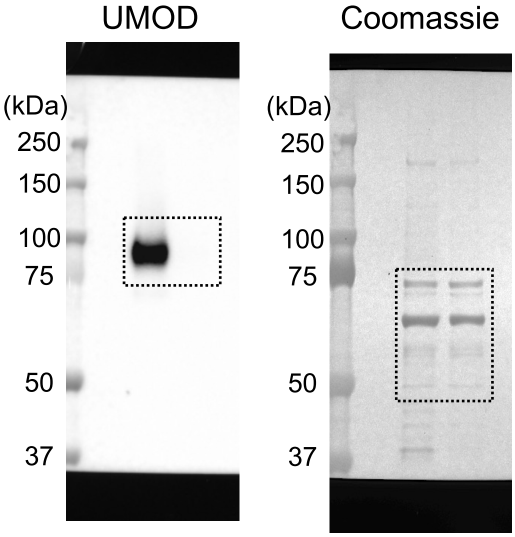

Figure 4A (C-UMOD expressing MDCK cells)

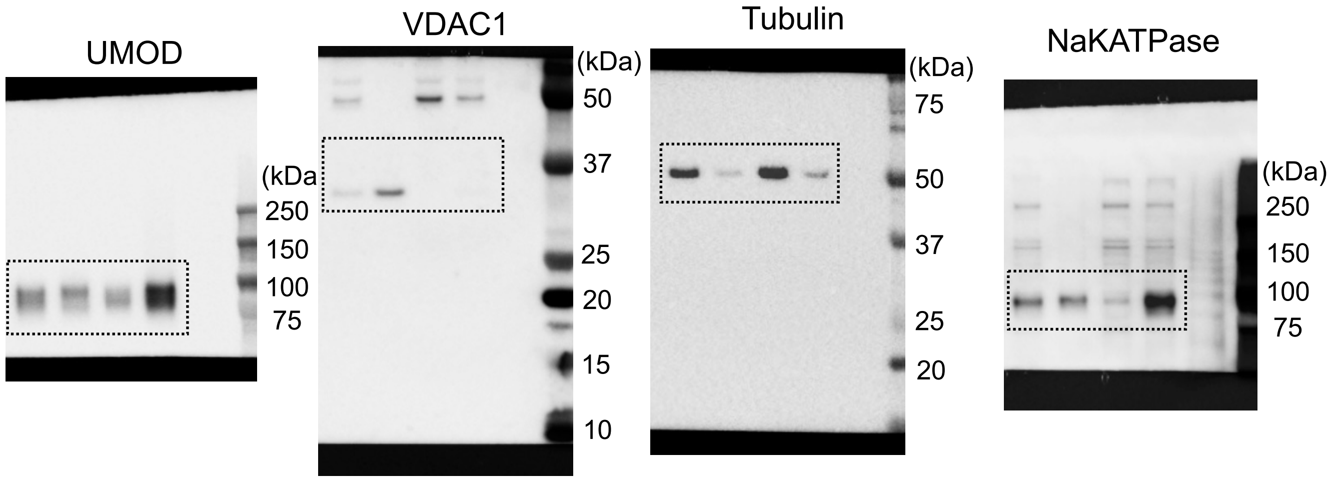

Figure 4A (AS-UMOD expressing MDCK cells)

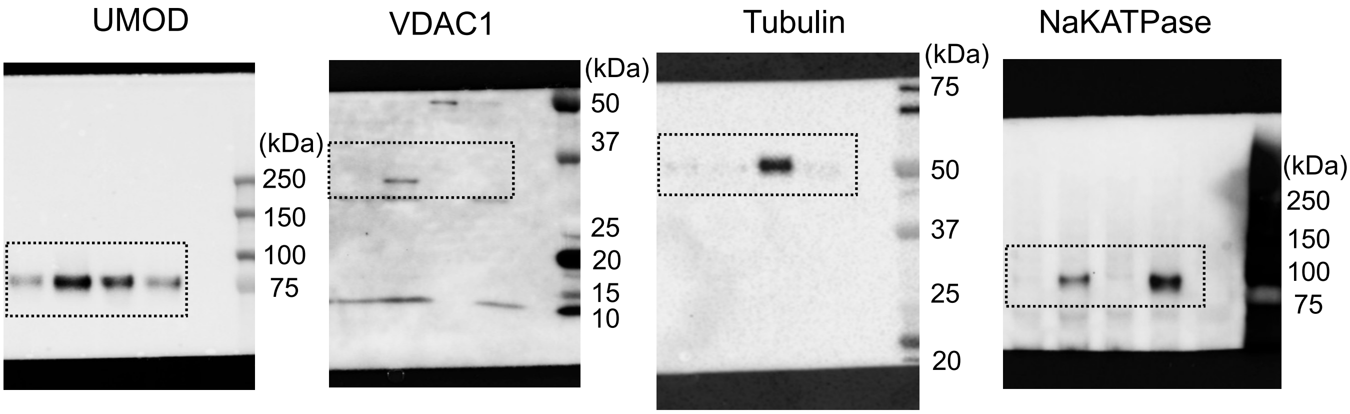

Figure 4H

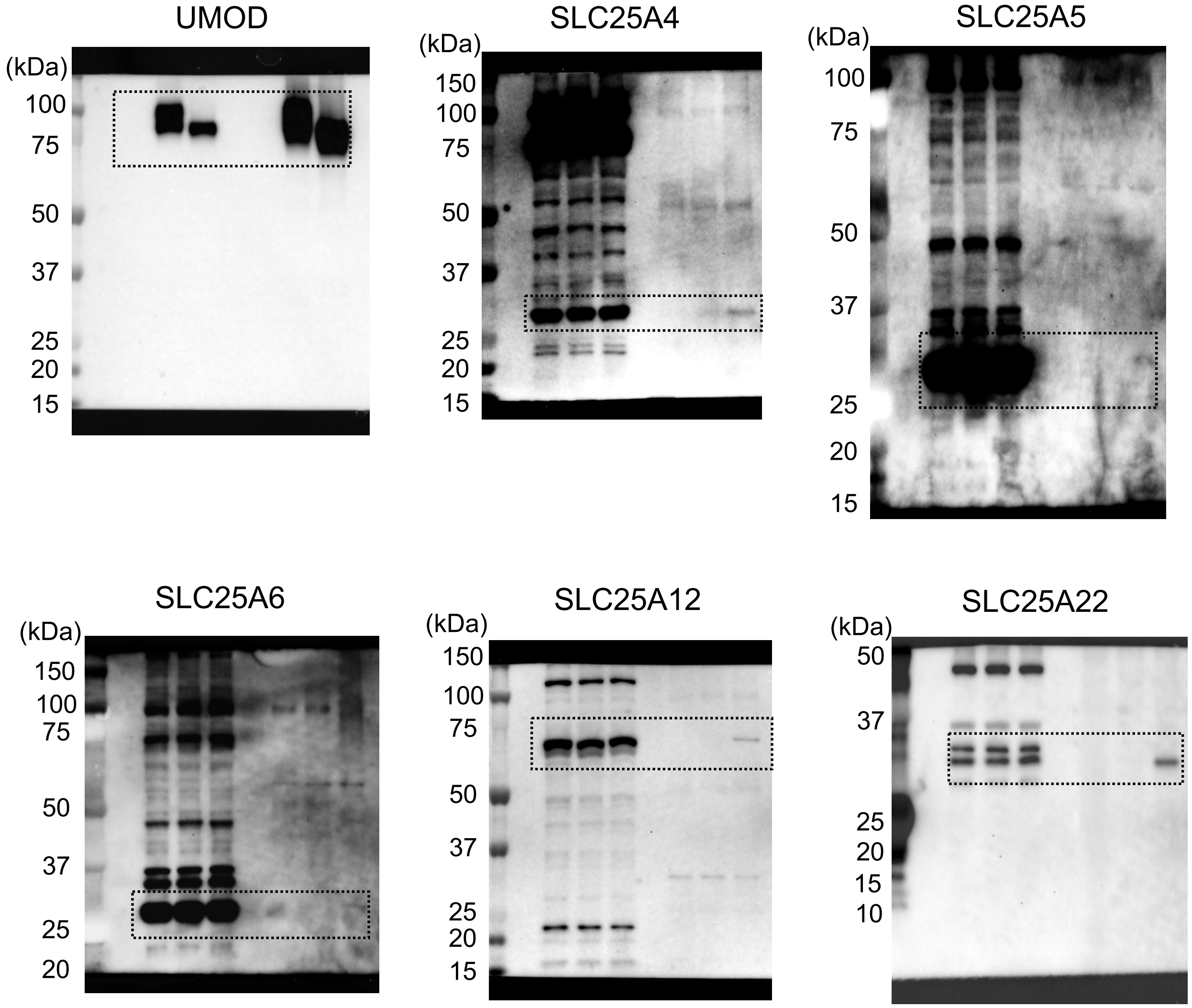

UMOD
(kDa)
100
75
50
37
25
20
15
SLC25A4
(kDa)
150
100
75
50
37
25
20
15
SLC25A5
(kDa)
100
75
50
37
25
20
15
SLC25A6
(kDa)
150
100
75
50
37
25
20
SLC25A12
(kDa)
150
100
75
50
37
25
20
15
SLC25A22
(kDa)
50
37
25
20
15
10

Figure 5B

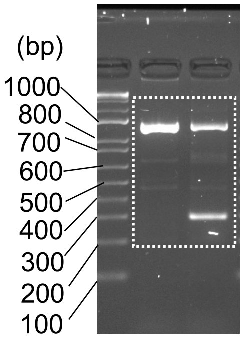

Figure 5E

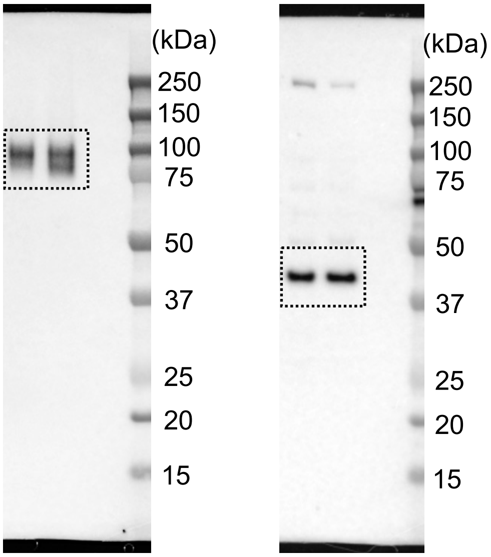

Figure 5F

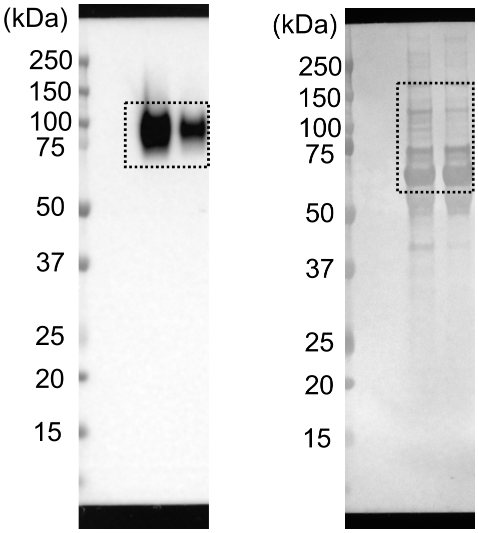

Figure 6A

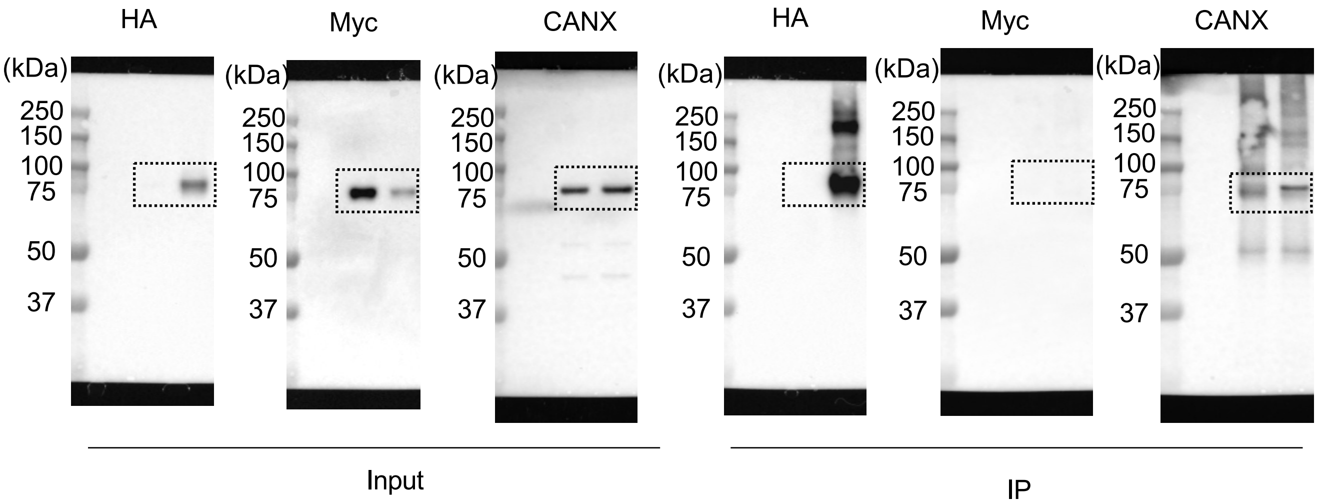

Figure 6B

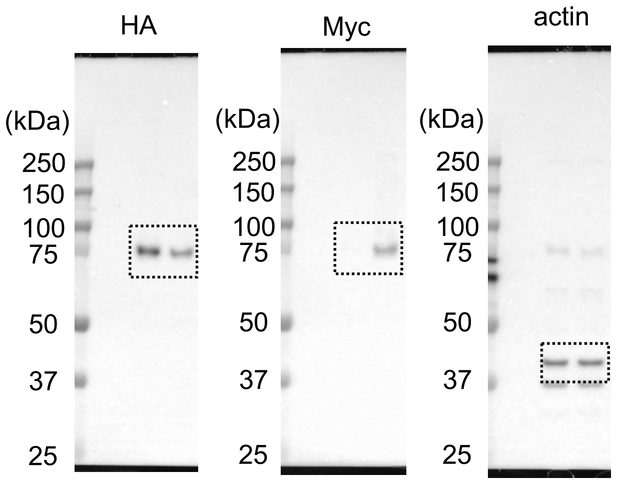

Figure 6C

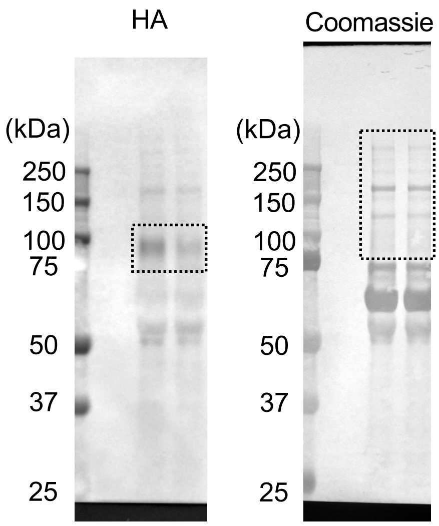

Figure 7D

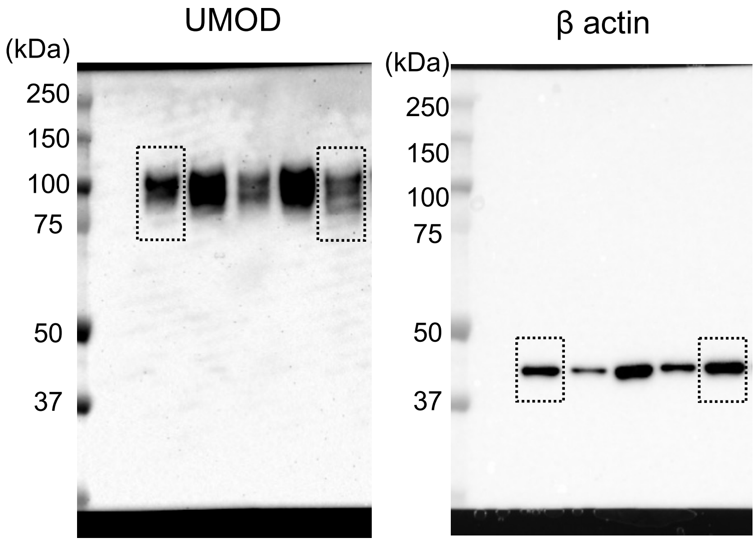

Figure 7E

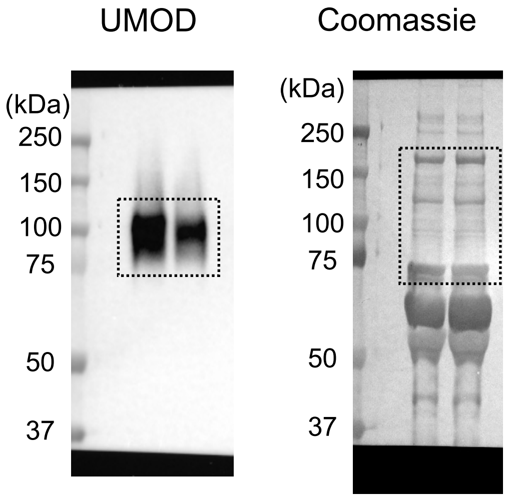

Supplementary Figure 6B

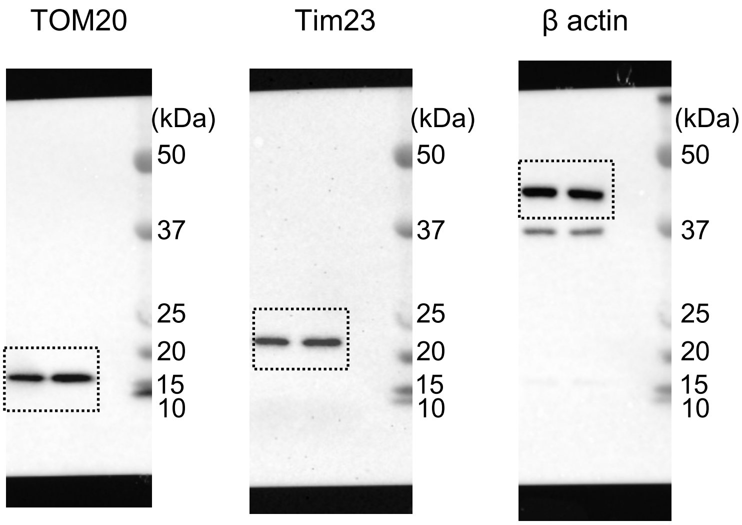

Supplement: Unedited blot and gel images [file jci-135-183343-s021.pdf]
